# A comprehensive gene expression analysis at sequential stages of *in vitro* cardiac differentiation from isolated MESP1-expressing-mesoderm progenitors

**DOI:** 10.1038/srep19386

**Published:** 2016-01-19

**Authors:** Sabine C. den Hartogh, Katherine Wolstencroft, Christine L. Mummery, Robert Passier

**Affiliations:** 1 Department of Anatomy and Embryology, Leiden University Medical Centre, Leiden, The Netherlands; 2 Leiden Institute of Advanced Computer Science Leiden Institute of Advanced Computer Science, Leiden University, The Netherlands; 3 Department of Applied Stem cell Technologies. MIRA Institute for Biomedical Technology and Technical Medicine. University of Twente, P.O.Box 217, Enschede, The Netherlands

## Abstract

*In vitro* cardiac differentiation of human pluripotent stem cells (hPSCs) closely recapitulates *in vivo* embryonic heart development, and therefore, provides an excellent model to study human cardiac development. We recently generated the dual cardiac fluorescent reporter MESP1^mCherry/w^NKX2-5^eGFP/w^ line in human embryonic stem cells (hESCs), allowing the visualization of pre-cardiac MESP1+ mesoderm and their further commitment towards the cardiac lineage, marked by activation of the cardiac transcription factor NKX2-5. Here, we performed a comprehensive whole genome based transcriptome analysis of MESP1-mCherry derived cardiac-committed cells. In addition to previously described cardiac-inducing signalling pathways, we identified novel transcriptional and signalling networks indicated by transient activation and interactive network analysis. Furthermore, we found a highly dynamic regulation of extracellular matrix components, suggesting the importance to create a versatile niche, adjusting to various stages of cardiac differentiation. Finally, we identified cell surface markers for cardiac progenitors, such as the Leucine-rich repeat-containing G-protein coupled receptor 4 (LGR4), belonging to the same subfamily of LGR5, and LGR6, established tissue/cancer stem cells markers. We provide a comprehensive gene expression analysis of cardiac derivatives from pre-cardiac MESP1-progenitors that will contribute to a better understanding of the key regulators, pathways and markers involved in human cardiac differentiation and development.

HPSCs provide an excellent platform to model human heart development and cardiac differentiation *in vitro*. We and others have previously shown that there are close similarities between the temporal and sequential transcriptional activation of genes during human cardiac differentiation *in vitro* and in cardiac development *in vivo*^1^,^2^. Identification of key molecular events during cardiac differentiation from human pluripotent stem cells is instrumental for a better understanding of human cardiac development, and advancing the fields of regenerative medicine, disease modelling, and drug discovery. Although cardiac differentiation protocols have been improved significantly over the last years, in-depth knowledge of molecular mechanisms involved in cardiac lineage commitment is sparse. Specification of hPSCs towards the cardiac lineage is regulated by the precise temporal expression of transcriptional networks. Detailed information on these transcriptional networks and signalling molecules is essential for understanding the mechanisms underlying progression and expansion of progenitors and their differentiation to their specific subtype derivatives. When hPSCs differentiate towards the cardiac lineage, they first progress via an intermediate mesodermal stage, expressing transcription factor MESP1, before they further differentiate into cardiac derivatives, indicated by the increased expression of cardiac marker NKX2–5^3^,^4^. In order to understand how molecular mechanisms play a role in these lineage decisions, we previously generated the dual cardiac human embryonic stem cell (hESC) reporter line MESP1^mCherry/w^NKX2-5^eGFP/w4^. We characterised MESP1 progenitors by gene expression and surface marker analysis, and showed that MESP1 derivatives were predominantly enriched for NKX2-5 positive cardiomyocytes. Here, we have defined the temporal gene expression changes that occur during the differentiation of isolated MESP1-progenitors towards their cardiac derivatives. By classifying enriched genes according to similar expression patterns, functional pathway analysis, GO terms, sequence-specific DNA binding properties, and protein-protein interactions, we provide more insight on molecular signals and regulators involved in the sequential stages of mesoderm to cardiac differentiation. We confirm the enrichment of previously identified transcriptional regulators and cardiac functional genes, indicated by the predominant activation of components of the Wnt and TGFβ pathways. Interestingly, we identified novel cardiac networks, signalling pathways and markers, which may have an important role in the early cardiac differentiation and may be used as blueprint to evaluate or study human cardiac differentiation *in vitro.*

## Results

### Collection of time points during cardiac differentiation

**Figure 1:**
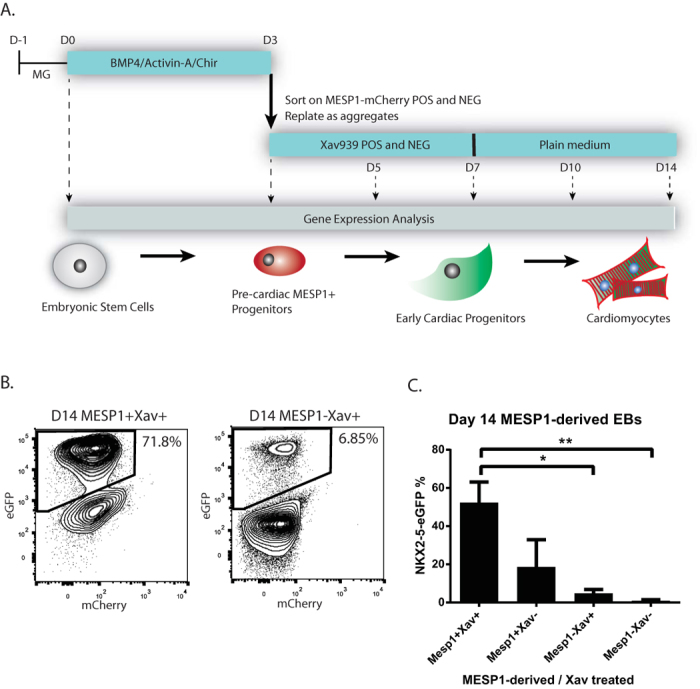
(**A**) hESCs were differentiated as monolayer and were sorted at day 3 on their MESP1-mCherry expression. Positive and negative fractions were further differentiated and RNA was collected at sequential timepoints day 5, day 7, day 10, and day 14. (**B,C**) The efficiency of the cardiac differentiations was monitored by flow cytometry at day 14 of differentiation. NKX2-5-eGFP levels were highly increased in the MESP1-positive derived fraction, treated with Wnt-pathway inhibitor Xav939. MESP1-negative fractions, lacking Xav939 treatment, were showing almost no NKX2-5-eGFP expression levels * P < 0.05, ** P < 0.001. Error bars indicate SEM: standard error of the mean. N = 3.

To obtain MESP1-mCherry progenitors for further differentiation, we differentiated and isolated MESP1^mCherry/w^NKX2-5^eGFP/w^ hESCs as described before^4^. Day 3 MESP1-mCherry positive and negative cells were replated as aggregates and further differentiated in the absence (control) or presence of Wnt-pathway inhibitor Xav939, which has been previously shown to enhance cardiomyocyte differentiation, (Fig. 1A). Aggregates were collected 2 (D5), 4 (D7), 7 (D10) and 11 (D14) days after sorting and RNA was isolated subsequently. Experiments were performed in triplicates. To ensure efficient cardiac differentiations in the samples that were used for gene expression analysis, flow cytometry analysis was performed at day 14 of differentiation from a similar differentiation experiment (Fig. 1B). We found 52.3 ± 10.7% of NKX2-5-eGFP positive cardiac cells in the MESP1-mCherry positive derived population, treated with Xav939, which was, as expected, significantly higher than 4.8 ± 2.0% in the MESP1-mCherry negative derived population, treated with Xav939 (Fig. 1C).

### Global gene expression patterns segregate upon cardiac differentiation

**Figure 2:**
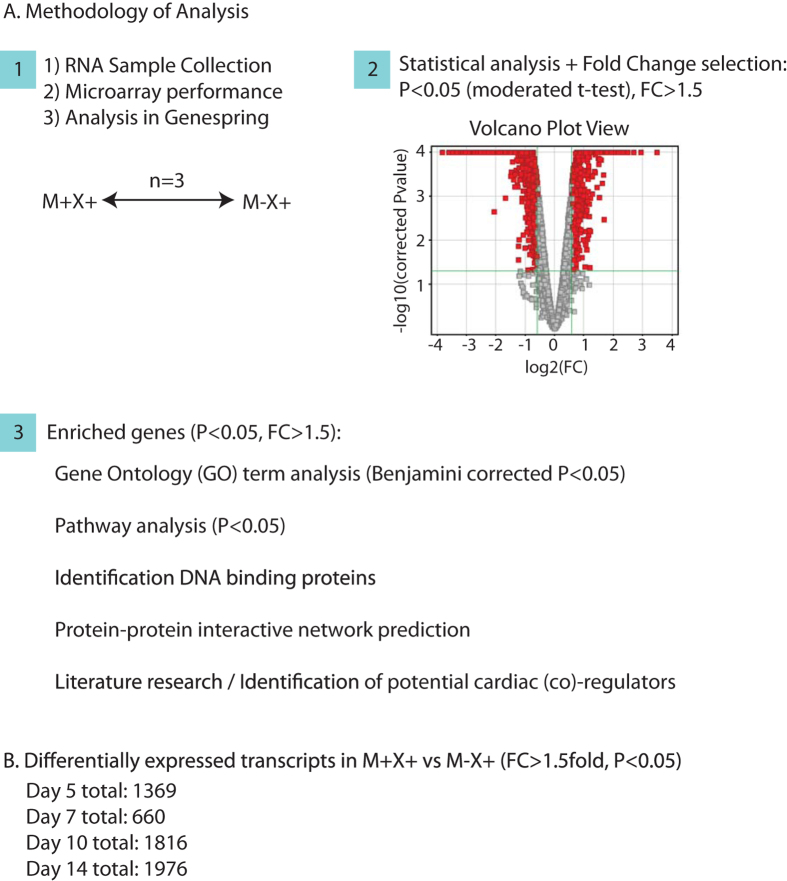
(**A**) Schematic overview of the methodology of gene selection from three biological replicate pairs, and the different methods of analysis that contribute to the identification of putative cardiac co-(regulators). A volcano plot view allows selection of differentially expressed genes, based on a P-value (P < 0.05, moderated t-test), and a fold change >1.5. (**B**) The number of differentially expressed transcripts at each timepoint (M+X+ vs M − X+), based on a FC > 1.5 fold difference, and a gene-specific p-value < 0.05. N = 3.

In order to study cardiac-specific enriched genes, we performed statistical analysis between replicates and generated gene lists based on a 1.5 fold change of expression level, with a gene-specific P-value < 0.05, in the MESP1-mCherry positive population, treated with Xav939 (M+X+), compared to the MESP1-mCherry negative population, treated with Xav939 (M − X+) (Fig. 2A). With this, we selected genes that displayed both large changes as well as more subtle, but consistent, changes^5^. The number of differentially expressed genes increased upon further differentiation (Fig. 2B), indicating a lower correlation between MESP1-positive and –negative samples upon further differentiation, and thus a more specialized gene expression pattern/phenotype. A top 100 list for both enriched and downregulated transcripts at each time point is given in Supplementary Table S1.

### Gene Ontology analysis shows a clear shift from global development to heart development

We determined which Gene Ontology (GO) categories were enriched for each specific differentiation stage, and could identify a clear shift between early cardiac progenitor stages D5 and D7, and the later cardiac-lineage-committed progenitors and cardiomyocytes (D10 and D14) (Table 1). D7 GO terms were highly similar to those in D5, which included GOs that were more broadly developmental related. D10 and D14 were both highly enriched for heart-specific regulatory and functional GO terms, including heart development (BP00251), and muscle contraction (BP00173). To note, GO analysis of downregulated genes at each timepoint (FC > 1.5 fold, P < 0.05), did not show any tissue-specific terms, and is therefore not shown.

### Transcriptional regulators during early cardiac commitment (D5)

**Figure 3:**
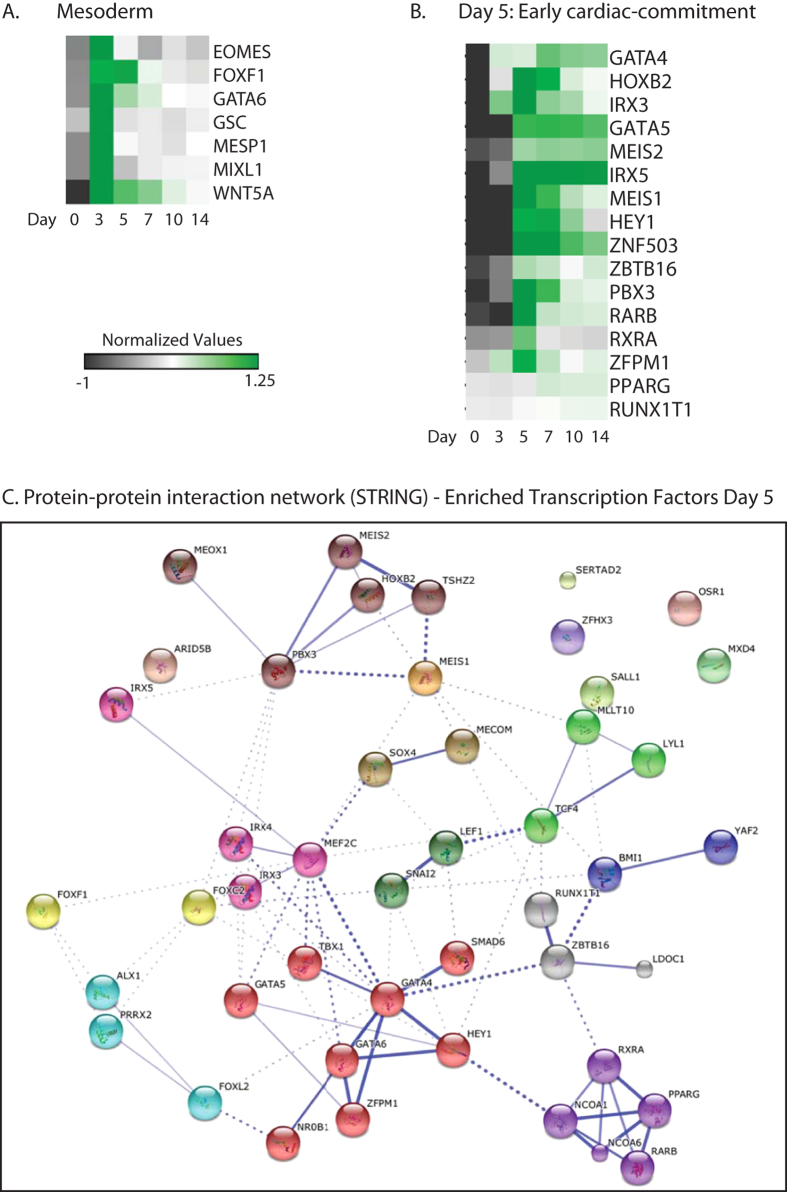
(**A**) Heatmap visualization of the relative expression levels of mesoderm genes throughout cardiac differentiation, showing a stage-specific enrichment in MESP1-mCherry isolated progenitors at day 3 of differentiation. Heatmap shows averaged values from n = 3. (**B**) Relative expression levels of DNA binding transcriptional regulators that were enriched at day 5 of differentiation in the MESP1-mCherry positive derivatives. Genes were clustered based on a One Minus Pearson Correlation. Heatmap shows averaged values from n = 3. (**C**) Evidence for protein-protein interaction networks of enriched transcription factors at day 5 of differentiation was constructed by STRING. Interactions with a medium confidence >0.4 are visualized. Proteins are clustered using the MCL algorithm. Every color represents a cluster. Inter-cluster edges are represented by dashed lines.

Firstly, in order to show that sorted MESP1-mCherry progenitors leave the mesoderm stage upon further differentiation, we generated an expression heatmap up to day 14 for a selection of genes that were previously demonstrated to be enriched in day 3 MESP1-mCherry positive progenitors^4^ (Fig. 3A). As expected, the levels of mesoderm transcription factors *MESP1, MIXL1, Eomesodermin (EOMES),* and *Goosecoid (GSC)* show a transient expression with a peak at day 3. In order to identify genes that may play important roles in early cardiac differentiation development we selected genes that were upregulated (FC > 1.5 fold, P < 0.05) in the day 5 M+X+ population, when compared to the day 5 M − X+ control. From the 281 enriched transcripts, (potential) cardiac (co)-regulatory genes were selected based on their predicted transcriptional activity, DNA binding domains, and biological function (Fig. 3B). Several transcription factors for which their role in early cardiac commitment has been shown previously could be identified based on their enrichment at day 5 of differentiation in the M+X+ samples (*GATA5, MEIS1, MEIS2, HEY1, IRX3, IRX5, GATA4,* and nuclear retinoic acid receptors *RARα* and *RARβ, and PPAR*γ). Some of these genes maintained high levels of expression at later stages. In order to identify novel cardiac regulators, we focussed on proteins with conserved DNA binding motifs, such as homeodomain and zinc-finger domains motifs. This list of genes included *HOXB2, ZFPM1, ZMBTB16, ZNF503,* and *RUNX1T1.* To understand how these genes and their encoded proteins could be involved in networks related to early heart development, we performed analysis using the STRING database for interactomic connections with established key transcription factors ( http://www.string-db.org/)^6^ (Fig. 3C). Using STRING, we predicted protein-protein associations based on *in vivo* and *in vitro* experimental assays, including gene co-occurrence in genomes (i.e. phylogeny), gene co-expression, gene fusion events, genomic neighbourhood (i.e. synteny), and experimental data such as co-immunoprecipitation and yeast two hybrid^6^.

We found a high predicted interaction between MEIS1, MEIS2, PBX3, and HOXB2, based on binding complexes of MEIS proteins with other PBX and HOX homologs in drosophila and rodent models^7^,^8^,^9^. Moreover, studies have indicated a crucial role for MEIS1, MEIS2, PBX3 and HOXB2 in either heart development, including heart looping and chamber septation^2^,^10^ or *in vitro* cardiac differentiation^2^,^11^. Interestingly, PBX3 has shown to induce either skeletal muscle in the presence of MyoD, a master regulator of skeletal muscle differentiation^12^,^13^, or cardiac differentiation, in the presence of the cardiac transcription factor Hand2^12^, indicating a crucial role for PBX3 as a cofactor during differentiation towards striated muscle. Moreover, MEIS1, MEIS2, HOXB2, and PBX3 were all upregulated upon Mesp1 induction in mouse ESCs, indicating that they act downstream of Mesp1^14^.

The genes *ZFPM1* (FOG1; friend of GATA family-1), *ZBTB16,* and *ZNF503* belong to the class of zinc finger transcription factors. FOG1 contains nine zinc-finger domains and belongs to a family of proteins of which two genes have been identified in mammals: FOG1 and FOG2. FOG proteins interact with the N-terminal domain of GATA factors and modulate their activity^15^ and have been shown to recruit nuclear receptor-transcriptional co-repressors and histone deacetylases (HDACs). Although the role of FOG1 in heart development is not well understood, one study in zebrafish showed the injection of an antisense morpholino directed against the homolog to murine FOG1 resulted in embryos with a large pericardial effusion and a deficient looping heart tube^16^.

Another zinc-finger domain protein that we found highly enriched in MESP1-positive derivatives at day 5, and that is also upregulated upon Mesp1 induction in mESCs^14^, is RUNX1T1 (runt-related transcription factor 1); a protein that is known to interact with transcription factors and to recruit a range of co-repressors to facilitate transcriptional repression^17^. In the human embryonic heart, RUNX1T1 expression is identified in both cardiomyocytes and endocardial cells^1^,^2^,^18^. Moreover, chromosome break points in the RUNX1T1 gene are associated with congenital heart disease^3^,^4^,^18^. Protein-protein interaction between RUNX1T1 and ZBTB16, a growth repressor in hematopoietic progenitor cells through its ability to recruit nuclear co-repressors such as histone deacetylases and Polycomb (PcG) family proteins, has been previously described^4^,^17^ and was therefore also predicted following analysis in the STRING database (Fig. 3C). Although no potential interactions in this cluster at day 5 were identified in the STRING database for Zinc Finger 503 (ZNF503), it has been previously classified as a potential human cardiac developmental regulator, based on its chromatin signature and its temporal expression level upon *in vitro* cardiac differentiation in hESCs^2^,^4^.

### Transcriptional regulators in early cardiac progenitors (D7)

**Figure 4:**
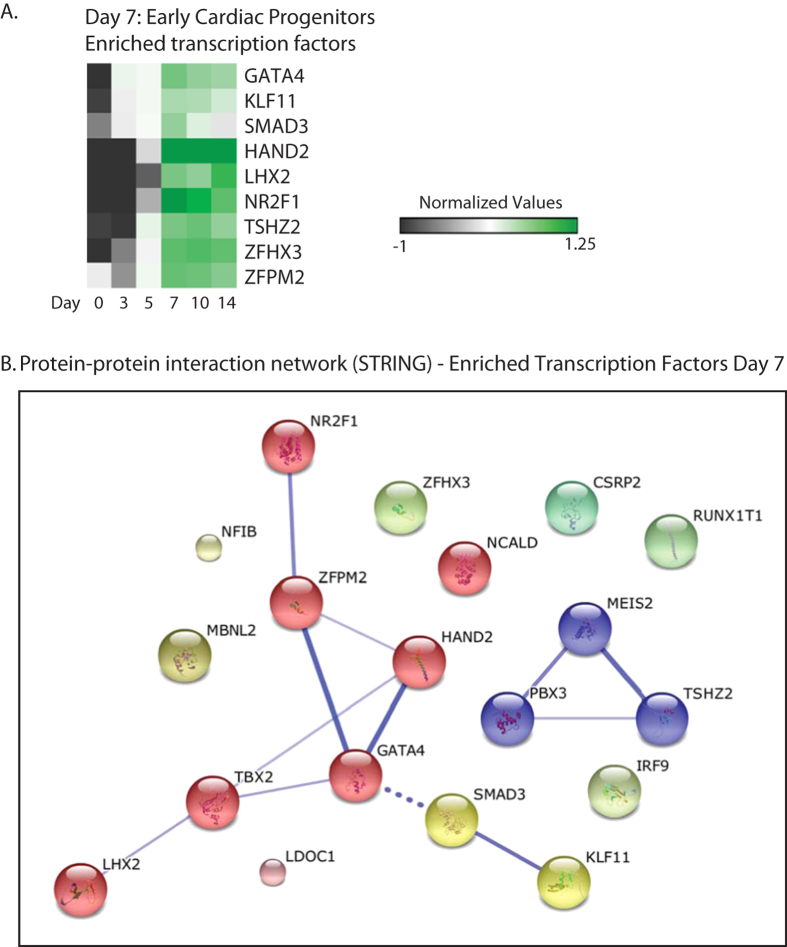
Enriched transcripts at day 7 of differentiation in MESP1-mCherry derivatives. Genes were selected based on a P-value > 0.05 and FC > 1.5 fold difference in expression, when compared day 7 MESP1-mCherry negative derivatives. (**A**) Enriched DNA binding transcripts at day 7 of differentiation in MESP1-mCherry derivatives. Heatmap shows averaged values from n = 3. (**B**) Evidence for protein-protein interaction networks of the enriched transcription factors at day 7 of differentiation was constructed by STRING. Interactions with a medium confidence >0.4 are visualized. Proteins are clustered using the MCL algorithm. Every color represents a cluster. Inter-cluster edges are represented by dashed lines.

Upon further differentiation of MESP1-derived cardiac committed cell lineages towards early cardiac progenitor stage (day 7 of differentiation), we found 660 differentially expressed genes (FC > 1.5 fold, P < 0.05) in day 7 M+X+ population, when compared to day 7 M − X+ control. Again, (potential) cardiac (co)-regulatory genes were selected based on their transcriptional activity, nucleotide binding domains, and biological function. We identified a subset of known and potential novel cardiac regulatory factors, including *GATA4, LHX2,* and *COUP-TF1 (NR2F1),* and DNA-binding zinc-finger proteins *ZFPM2 (FOG2), TSHZ2,* and *ZFHX3* (Fig. 4A).

STRING-based interaction analysis of genes enriched at day 7 showed predictive interactions of FOG2 with COUP-TF1 and GATA4 (Fig. 4B). It has been previously shown that FOG2 represses COUP-TFII dependent synergistic activation of the atrial natriuretic factor promoter, suggesting that FOG2 functions as a co-repressor for both GATA and COUP-TF proteins^5^,^19^,^20^, although it may also act as a co-activator in combination with other transcription factors^4^,^20^,^21^. Zinc finger homeodomain protein 3 (ZFHX3) contains 4 homeobox domains and 22 zinc finger domains, and is described as transcriptional repressor for myogenic differentiation through repression of the *MYF6* gene^6^,^22^. Based on chromatin signature and transient expression levels during *in vitro* cardiac differentiation in hESCs a regulatory role for ZFHX3 in human cardiac development has been suggested^2^,^6^. Moreover, sequence variants of ZFHX3 are associated with atrial fibrillation^7^,^8^,^9^,^23^,^24^,^25^. The zinc finger transcription factor TSHZ2, may be a potential transcriptional repressor for MEIS transcription factors (Fig. 4B), based on interaction studies between conserved orthologs in drosophila. Its role in heart development is currently unknown.

### Expression profile plots of potential regulatory genes show a distinct expression pattern throughout cardiac differentiation

**Figure 5:**
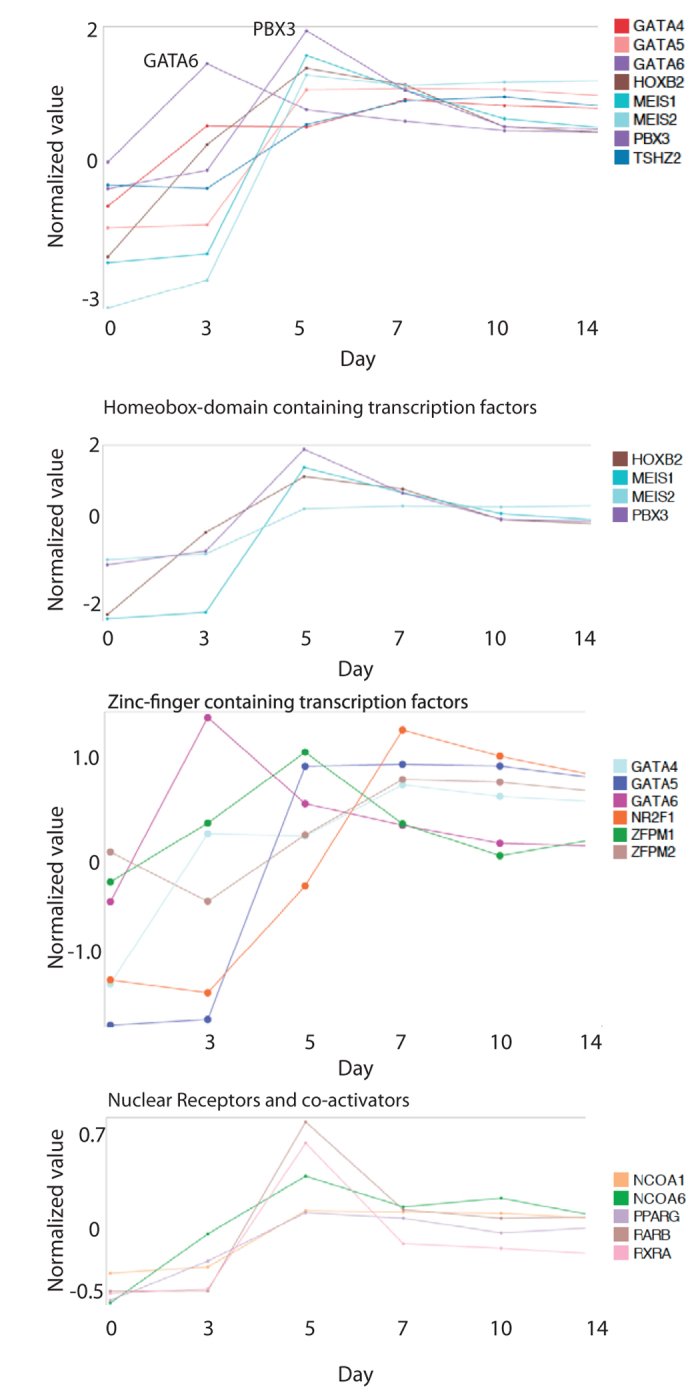
Profile Plots of Day 5 and Day 7 Enriched Transcription Factors. The upper panel shows the expression patterns upon cardiac differentiation of a selection of DNA binding transcription factors. The second upper panel shows the similarities between the expression patterns of HOXB2, MEIS1, MEIS2, and PBX3. The second last panel shows the expression pattners of GATA factors and ZFPM factors, and shows the similarities between ZFPM2 and GATA4 from day 7–14 of differentiation. The lower panel shows the expression levels of retinoic acid nuclear receptors and their co-activators, peaking at day 5 of differentiation. Normalized alues are visualized on a global expression scale and averaged from n = 3 at each timepoint.

Stage-specific (co)-expression of transcriptional genes could indicate a regulatory role. Here, we show how similarities between expression profiles of PBX3, MEIS1, MEIS2, and HOXB2 throughout cardiac differentiation further strengthen the hypothesis that they act in a similar stage-specific molecular role, potentially as co-regulators (Fig. 5). In addition, similar expression profiles of GATA4 and FOG2 during cardiac differentiation support a potential co-regulatory role (Fig. 5). Further, we show how expression levels of TSHZ2 distinctly increase throughout cardiac differentiation from day 5 onwards, suggesting a role for TSHZ2 in cardiac differentiation (Fig. 5).

### Gene expression profiling of MESP1-derived cardiac cells (D10/D14)

**Figure 6:**
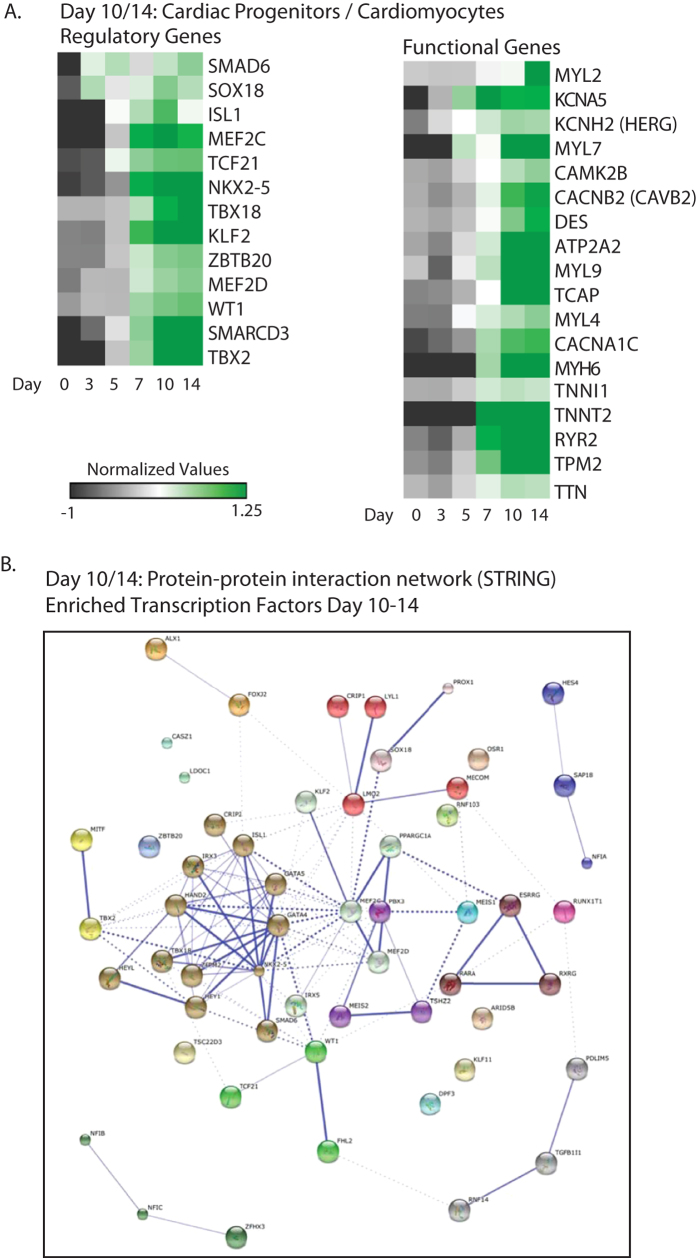
Enriched transcripts at day 10 and day 14 of differentiation in MESP1-mCherry derivatives. Genes were selected based on a P-value > 0.05 and a FC > 1.5 fold difference in expression with day 10 and day 14 MESP1-mCherry negative derivatives. (**A**) A selection of regulatory and functional genes that were enriched. Heatmaps show averaged values from n = 3. (**B**) Evidence for protein-protein interaction networks of the enriched transcription factors at day 10 and 14 of differentiation was constructed by STRING. Interactions with a medium confidence >0.4 are visualized. Proteins are clustered using the MCL algorithm. Every color represents a cluster. Inter-cluster edges are represented by dashed lines (C).

Upregulated genes at day 10 (952) and day 14 (1062) of differentiated samples from M+X+ were classified as described before and expression levels were visualized in heatmaps (Fig. 6A). Several genes from this list are known to play a key role in cardiac development, such as *NKX2-5, TBX18, WT1, TCF21, TBX2, HAND2, MEF2C, ISL1,* and *SMARCD3.* Interaction analysis of all transcription factors enriched in day 10 and day 14 samples showed a distinct regulatory cluster centred on NKX2-5 and GATA4 (Fig. 6B). The presence of highly enriched levels of cardiac genes encoding for structural and sarcomeric proteins and ion channels indicates the presence of functional cardiomyocytes (Fig. 6A). Moreover, while most structural genes are increased at cardiac progenitor stage day 7, levels of ventricular marker MYL2 could only be identified in clusters of upregulated genes at day 14. This is in agreement with previous studies, showing that MYL2 expression is only highly increased after approximately one month of cardiac differentiation^2^,^10^,^26^, suggesting that *MYL2* may serve as a marker for cardiomyocyte maturation.

Further, we could identify a node cluster of TCF21 and WT1, connected to TBX18; three regulatory transcription factors implicated in the formation of epicardial progenitors, indicating either the presence of these progenitors in our MESP1-derived cardiac aggregates or the potential to further differentiate to epicardial cells^2^,^11^,^27^. Furthermore, we identified enriched levels of growth factors VEGFA/B/C and receptor Neuropilin-1 (NRP1), ANGPT1, and surface marker CD34, indicating the derivation of vascular endothelial progenitors from MESP1 progenitors (Fig. S1).

### Merging transcriptional networks sequentially active throughout cardiac differentiation

**Figure 7:**
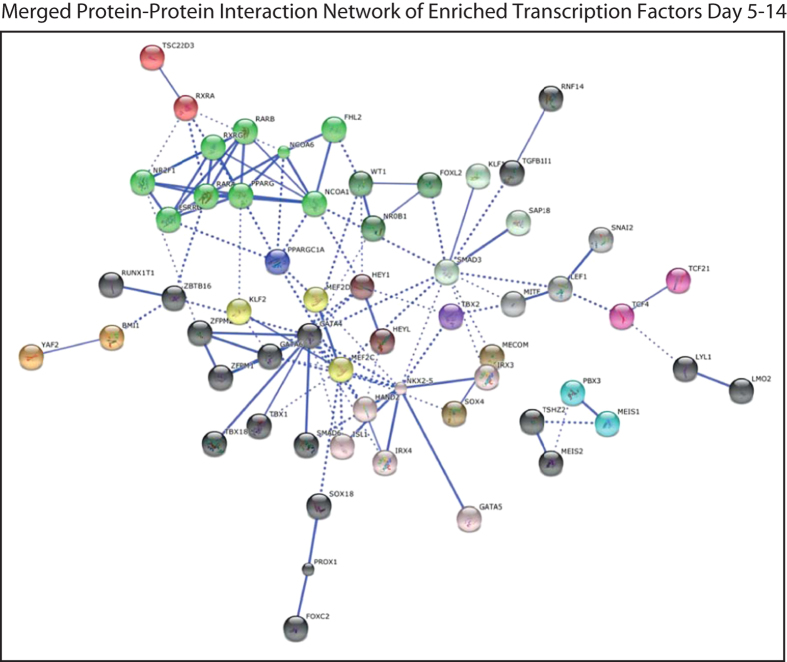
Merged protein-protein interaction network of the enriched transcription factors from all timepoints (d5, d7, d10, d14), obtained from STRING. Interactions with a high confidence >0.7 are visualized. Proteins are clustered using the MCL algorithm. Every color represents a cluster. Inter-cluster edges are represented by dashed lines. Non-connected gene nodes are not visualized. We could identify the development of three distinct large node clusters, including retinoic acid nuclear receptors, containing PPARγ and RAR α/β, and two large interconnected cardiac networks centered on either GATA4 or NKX-5.

In order to understand how cardiac transcriptional networks may be developed along cardiac lineage commitment and differentiation we generated a large interactive network, based on transcription factors that are enriched throughout all timepoints of differentiation (obtained through Gene Set Enrichment Analysis (GSEA) from Broad Institute) (Fig. 7). Interestingly, we could identify the development of three distinct large node clusters, including retinoic acid nuclear receptors, containing PPARγ and RAR α/β, and two large interconnected cardiac networks centered on either GATA4 or NKX2-5.

### Signalling pathways during cardiac differentiation

**Figure 8:**
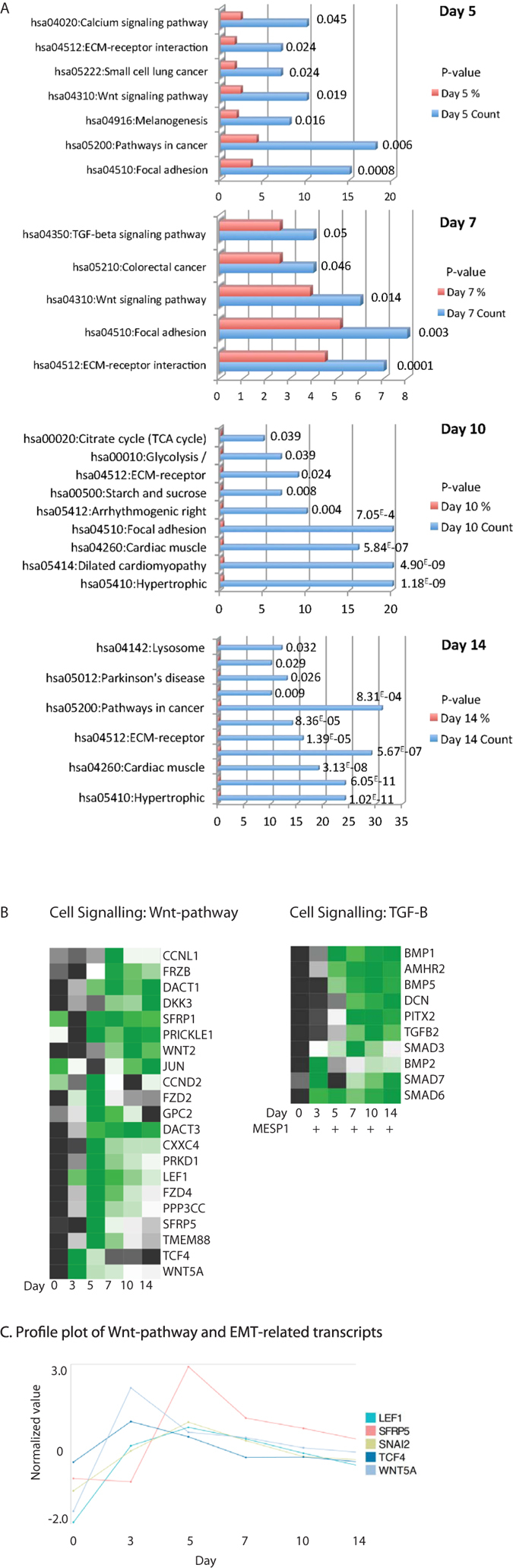
Enriched signalling pathways were identified through KEGG pathway analysis. (**A**) Pathway analysis was performed on the enriched genes, selected as described before. Pathways that are visualized have a P < 0.05. Gene count indicates the number of genes that could be identified for each enriched pathway. % indicates the percentile of the gene count from the total number of genes in the specific pathway. Numbers indicate the pathway-specific p-value. A comprehensive list of pathways can be found in Table S2. (**B**) Heatmap visualization of the enriched transcripts belonging to the Wnt signalling pathway, which is enriched in early cardiac committed progenitors, and the TGF-β signalling pathway, enriched in cardiac progenitors and cardiomyocytes. Heatmaps show averaged values from n = 3. (**C**) Profile Plots of enriched Wnt-pathway-related and EMT-related gene expression levels throughout cardiac differentiation. Normalized values are visualized on a global expression scale and averaged on n = 3.

In order to study the role of specific signalling pathways during cardiac lineage differentiation, we performed pathway analysis using the KEGG pathway database source (Fig. 8A, Supplementary Table S2) on the enriched genes at each stage of differentiation (M+X+ vs M − X+, FC > 1.5 fold, P < 0.05). From these results, we could clearly find an important role for Wnt-signalling pathway components during early cardiac lineage commitment (Day 5, Day 7), corroborating the findings from our previous study, in which we demonstrated non-canonical enrichment of Wnt pathway molecules, including Wnt5a and Wnt inhibitors DKK1 and DKK4 in MESP1 expressing progenitors^4^,^12^,^13^. Figure 8B shows an expression heatmap for enriched Wnt-pathway related genes across all time points (Fig. 8B), including SFRP5, SFRP1, FZD2, FZD4, and WNT5A, TCF4, and EMT-regulators SNAI2, and LEF1 (Fig. 8C). Interestingly, we identified significant enrichment of Wnt/β-catenin antagonists, including SFRP5, WNT5A, DACT1, and DACT3, in particular temporally peaking at early time points of cardiac differentiation, which would lead to downregulation of Wnt/β-catenin signalling, necessary for cardiac specification (Fig. 8C)^12^,^28^. Furthermore, through pathway analysis and protein-protein interaction analysis using STRING, we identified a prominent role for TGF-β signalling pathway components in early cardiac progenitor stage and cardiomyocytes (d7–d14) (Fig. 8B, Supplementary Table S2). TGF-β signalling is important for cardiovascular development and plays an important role in epithelial-to-mesenchymal transition (EMT), in order to stimulate endocardial and epicardial transitions to mesenchymal cells of the heart^14^,^29^. Expression analysis of a selection of these transcripts demonstrates a similar profile, suggesting interaction and or co-regulation of Wnt/β-catenin antagonists, Wnt/β-catenin target element TCF4, and EMT-related transcription factors SNAI2 and LEF1, which can be activated by both Wnt/β-catenin and TGF-β signalling (Fig. 8C)^30^.

### Role of ECM proteins during cardiac differentiation

**Figure 9:**
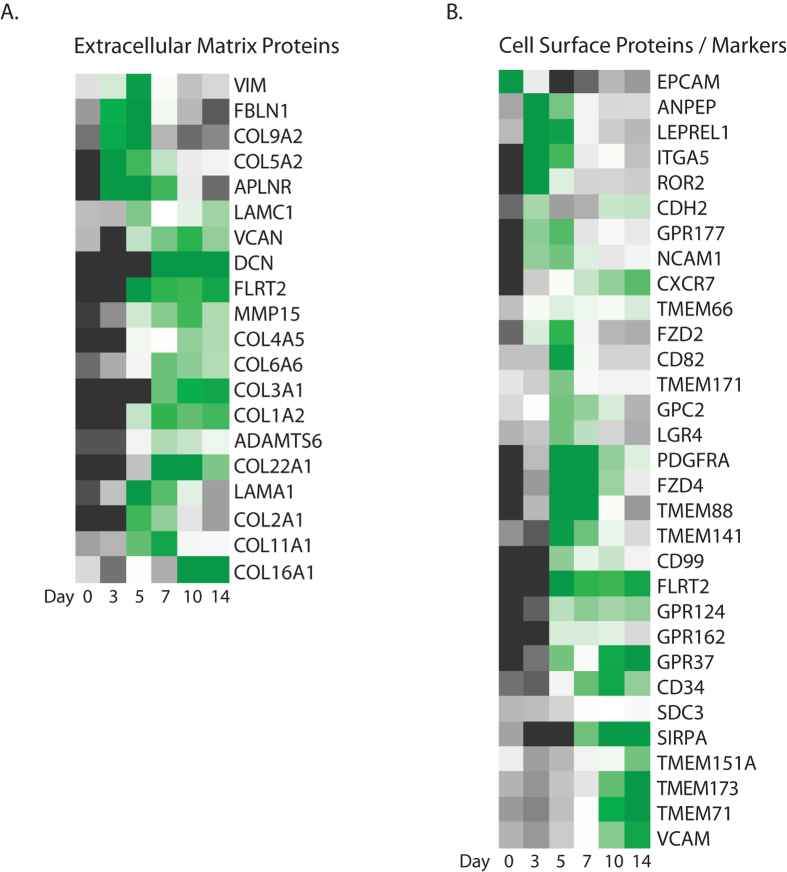
(**A**) Heatmap visualizes the temporal enrichment of extracellular matrix proteins throughout cardiac differentiation. (**B**) Visualization of temporal expressed cell membrane proteins to identify potential cell surface markers for subsets of cardiac progenitors or cardiomyocytes. Heatmaps show averaged values from n = 3.

Extracellular matrix (ECM) proteins play an important role in the formation of the microenvironment for cells and provide different stimuli leading to activation of numerous molecular and cellular mechanisms, including migration, proliferation and differentiation of cells, as these events take place during the different stages of cardiac development^15^,^31^,^32^. Pathway analysis of enriched genes in the MESP1-derivatives indicated an important role of ECM signalling upon formation of the early cardiac progenitor populations and in established cardiomyocytes (Fig. 8A). Interestingly, the composition of this cluster showed dynamic changes throughout cardiac differentiation (Fig. 9A), suggesting the need for a specific composition of proteins forming a microenvironment or niche for cardiac progenitor cells and cardiomyocytes for optimal functioning in processes such as self-renewal, differentiation, and survival^16^,^32^,^33^. Moreover, we identified also other transiently upregulated levels of ECM components and proteases that have been implicated in heart developmental defects^14^,^32^ (Fig. 9A).

### Surface marker expression analysis on MESP1-derived cardiac progenitors

An efficient approach to isolate specific subpopulations for translational applications will be the use of antibodies against distinct cell surface markers. For this, we analysed expression patterns of a large variety of cell surface markers that were enriched in the MESP1-derived cardiac populations at the different time points (Fig. 9B). In our previous study we found that cell surface markers N-cadherin (CDH2), CD13 (ANPEP), and ROR2^4^,^17^ are broadly expressed in mesodermal cells and not specifically for MESP1-expressing progenitors. Surface proteins that show a transient peak expression at day 5 (M+X+), include TMEM88, CD82, TMEM171, LGR4, and CD74. From these, only TMEM88 has been clearly associated with heart development and acts downstream of GATA factors in the pre-cardiac mesoderm to specify lineage commitment of cardiomyocyte development through inhibition of Wnt/β-catenin signaling^34^,^35^. Interestingly, LGR4 belongs to the Leucine-rich Repeat-containing G protein-coupled receptor family with a close relation to LGR5 and 6, known stem cell marker for different tissues. LGR4 is expressed in the heart^36^,^37^, although not exclusively, but no role has been described so far for heart development or cardiac stem cells. Surface markers that show high enrichment at day 5 of differentiation, with a continuous enrichment upon further cardiac differentiation (up to final cardiomyocyte formation) include GPC2, FZD4, PDFGRα, NCAM1, FLRT2, TMEM66, and GPR124. We, and others, have shown that PDFGRα and NCAM1 broadly mark mesoderm progenitors^4^,^38^. In early embryonic development, Fzd4 is highly expressed in the cardiac crescent, head mesenchyme, and later in the developing heart tube^39^. Loss of Fzd4 shows a decrease in density and branching of small arteries in the developing mouse heart^40^. Flrt2 and Gpr124 could both be potential markers for cardiac lineage-commitment. Flrt2 is abundantly expressed in developing mouse heart tissue and loss of Flrt2 results in in impaired expansion of the compact ventricular myocardium^41^. G-protein coupled receptor *GPR124* shows a similar stage-specific expression pattern as *FLRT2*. However, *GPR124* is expressed on endothelial cells during blood vessel development, but not specifically expressed in the heart^42^. Transmembrane protein 66 (TMEM66) functions as calcium ion transmembrane transporter, and is enriched in MESP1 progenitors and their cardiac derivatives up to day 14. However, *in vivo* it has been shown that TMEM66 is not specific for the developing heart^43^.

Surface protein transcripts that are specifically enriched in late cardiac progenitors (day 10), and cardiomyocytes (day 14) include *TMEM151A, VCAM1, TMEM71, TMEM173*, and *SIRPA.* VCAM1 and SIRPA have been previously identified to be specific human CM markers^3^,^44^,^45^. Mouse embryo expression databases describe TMEM71 in mesoderm tissues, including atria and ventricles. Not much is known about TMEM151A and TMEM173 expression in the heart.

## Discussion

For studying the temporal expression of key molecules involved in cardiac lineage commitment upon differentiation, we recently generated the dual cardiac fluorescent reporter MESP1^mCherry/w^NKX2-5^eGFP/w^ hESC line, allowing us to isolate early pre-cardiac mesoderm progenitors and follow their further differentiation towards NKX2-5 expressing cardiomyocytes^4^. In the current study, we used this dual cardiac reporter line to perform molecular profiling during cardiomyocyte differentiation from MESP1-expressing pre-cardiac progenitors by genome-wide transcriptomic analysis encompassing crucial events during cardiac differentiation from hPSCs: early cardiac lineage commitment, early cardiac progenitor stage, late cardiac progenitor stage, and functional cardiomyocytes. Global gene expression analysis and gene ontology analysis confirmed and visualized that expression patterns became more specified towards cardiac differentiation, shifting from developmental networks to cardiovascular-specific developmental networks. Throughout differentiation, we identified sequential expression patterns of key cardiac transcription factors, followed by enriched levels of structural and functional cardiac genes at day 10 and day 14 of differentiation, indicating that in the presence of Wnt-pathway inhibitor Xav939, isolated MESP1 progenitors have the preference to differentiate to the cardiac lineage. Using pathway analysis we identified a prominent role for Wnt-pathway antagonists during early cardiac lineage commitment leading to inhibition of Wnt/β-catenin signalling. Furthermore, using STRING software^6^ that builds functional protein-association networks based on compiled available experimental evidence, we identified a subset of transcription factors during early lineage commitment: *HOXB2, ZFPM1, ZBTB16, ZNF503, RUNX1T1,* ZFPM2, and ZFHX3. Zinc-finger proteins ZFPM1 (FOG1) and ZFPM2 (FOG2) belong the FOG (friend of GATA) gene family, however, their role in heart development has not been elucidated yet. Based on previous studies, FOG proteins interact with GATA and COUPTF proteins, key transcription factors for early developmental processes, including heart development and lineage commitment^19^,^20^,^21^. FOG1 and FOG2 are indicated as co-activators or co-repressors of GATA- and COUPTF-activity on downstream cardiac genes, dependent on the transcriptional network that is active, pointing towards a putative role in cardiac lineage commitment. Besides their possible role in cardiac differentiation, FOG1 is expressed in blood islands of the yolk sac in mice and act as a cofactor with GATA1 to induce transcriptional activity of downstream genes in erythroid and megakaryocytic cell differentiation^46^,^47^, which may indicate the presence of MESP1-derived hematopoietic progenitors in our cultures^48^,^49^. Interestingly, FOG1 antagonizes GATA-1 activities in other cell lineages^46^. Repression of other lineages may also lead to a preferred induction of the cardiac lineage. Here, we did not found an enrichment of GATA-1, nor did we find enrichment of surface receptor KDR, or ETV2 and Tal1, transcription factors that are important for a MESP1-progenitor derived hematopoietic lineage^48^. In contrast, we did find increased expression of GATA4, 5 and 6 at this stage of development, which could indicate a potential binding of FOG1 to the conserved amino zinc finger of these factors^15^,^20^.

Furthermore, homeodomain protein HOXB2 has been previously identified as potential cardiac regulator, based on its epigenetic signature^2^, although a more extensively investigated role for HOXB2 in anterior-to-posterior patterning has been described in hindbrain development^7^,^11^. In our study, we found temporal stage-specific co-expression of HOXB2 with PBX3, MEIS1 and MEIS2. STRING software showed binding evidence between these genes, based on experimental evidence of heterodimeric transcription complexes between PBX, HOX, and MEIS family genes^7^. Although a role for this predicted complex has not yet been described in the heart, both PBX3 and MEIS1 and MEIS2 have been implicated in heart development.

Network analysis revealed another cluster of transcription factors, consisting of RUNX1T1 and ZBTB16, which were first identified at day 5 of differentiation but maintained high expression levels throughout the course of cardiac differentiation. RUNX1T1 is described as a co-repressor for ZBTB16, important for hematopoietic lineage differentiation^17^. It is not clear whether these factors may also have a role during early cardiac differentiation, since increased expression is maintained throughout cardiac differentiation, or whether absence of other key regulators of hematopoietic differentiation prevented a hematopoietic molecular profile. Similarly, zinc finger transcription factor ZNF503, described during hindlimb and brain development, may also have a role in cardiac differentiation, since peak expression was observed at day 5 with continued expression levels throughout differentiation. ZFHX3 is another zinc finger homeobox domain containing protein that finds enriched levels in cardiac progenitors and cardiomyocytes. SNP variants in the coding sequence have been correlated to atrial fibrillation, suggesting a role in heart development^23^,^24^.

Furthermore, we studied the temporal expression patterns of enrichment cell surface markers that could be useful for efficient cardiac progenitor and/or cardiomyocyte isolation experiments. Several cell surface markers have been identified including LGR4, which is an R-spondin receptor with strong positive effect on Wnt signalling, and could play a role in self-renewing capacity of early cardiac progenitors^50^,^51^. Its homologs LGR5 and LGR6 are well-known stem-cell-growth markers in other organ stem cells, including that of the intestine, stomach, and hair-follicle^52^, which makes the role and expression of LGR4 in cardiac progenitors of high interest to study. Furthermore, we identified other cell surface markers, and found that transmembrane protein transcripts *TMEM151A, TMEM71*, and *TMEM173* show a stage-specific enrichment similar to that of *SIRPA* and *VCAM1,* both human-specific cardiomyocyte markers^44^.

Another key finding in this paper is the importance of ECM components upon cardiac differentiation. For example, we find transcripts as COL9A2 temporal-specific enriched at day 5 of differentiation, where a large number of other collagen transcripts are enriched throughout complete cardiac differentiation. Similar stage-specific patterns are seen for other ECM proteins.

A complete understanding of how ECM proteins are involved in heart development is lacking. Increasing our knowledge of stage-specific ECM-controlled steps in early heart development will be valuable for understanding pathology of diseased hearts.

Furthermore, the variety of enriched cardiovascular transcripts in day 10 and day 14 M+X+ samples, including HAND2, which is expressed throughout the heart predominantly in the right ventricle; epicardial progenitor transcription factors *WT1, TCF21,* and *TBX18;* and vascular endothelial progenitor surface marker CD34, vascular growth factors VEGFA/B/C, and cell surface receptor tyrosine kinase (TEK/TIE2), which is required for normal angiogenesis and heart development during embryogenesis, indicate the mixture of cell populations that derives from MESP1 progenitors, or the requirement of these transcripts for optimal cardiomyocyte differentiation cultures.

To conclude, results from our comprehensive gene expression analysis revealed several potential novel cardiac regulators, either as (co)-activator, or (co)-repressor, dependent on the transcriptional network active. Future studies will clarify the role of these identified factors during early cardiac differentiation and whether they have multiple roles during lineage. In addition, besides their role in lineage specification, these factors may also play additional roles in other cellular processes such as proliferation, differentiation, or cell survival. Therefore, to further validate the role of predicted regulatory genes in early human heart development and/or in the onset of congenital heart disease, experimental interaction analysis, direct downstream gene target analysis, and knockdown studies *in vivo* and *in vitro* will be required.

## Methods

### hESC maintainance, cardiac differentiation, and sample collection

MESP1^mcherry/w^NKX2-5^eGFP/w^ hESCs were cultured and differentiated as described before^4^. In brief, hESCs were induced with BMP4 (30 ng/mL, R&D Systems), Activin A (20 ng/mL, Miltenyi Biotec), and Chir99201 (1.5 μM, Axon Medchem). At day 3 of differentiation, MESP1-mCherry positive and negative progenitors were isolated by FACS and aggregated for further differentiation, in the presence of 5 μM Xav939 (R&D). Total RNA was isolated using Nucleospin RNA XS kits (Macherey Nagel), at day 5 (2 days after replating), day 7, day 10, and day 14 of differentiation (Fig. 1). At day 14 of differentiation, samples were analysed on NKX2-5-eGFP expression by flow cytometry, as described before^4^.

### Gene expression micro array and data analysis

RNA quality control, RNA labeling, hybridization and data extraction were performed at ServiceXS B.V. Microarray analysis was performed on three biologically independent replicates using Illumina human HT‐12v4 arrays (ServiceXS B.V.). Data analysis was performed using Genespring (Agilent Technologies). Previously published micro array data that was complementary used in this study is numbered as: GSE73651. First, to filter probe sets on outlying values, we performed a one-ANOVA significant analysis test. P-values were corrected using the Benjamin-Hochberg method (corrected P-value < 0.05). In order to select enriched genes in MESP1-mCherry positive derivatives compared to MESP1-mCherry negative derivatives, we performed statistical analysis, using a moderated T-test, on normalized values from three biological replicates. P-values were corrected using the Benjamin-Hochberg method. By using a volcano plot view, enriched genes were selected by FC > =1.5 fold value difference in MESP1-mCherry positive derivatives compared to MESP1-mCherry negative derivatives, with a P-value < 0.05 (Fig. 2). GO analysis was performed on the selected genes, with a multiple-GO-term correction using the Benjamini-Yekutieli method, and a P-value cut-off of P < 0.05. For GO analysis we used the DAVID Bioinformatics Recources 6.7 database from NIH ( https://david.ncifcrf.gov/). Pathway analysis on the FC > =1.5 fold selected genes was performed using KEGG pathway databases. Pathways with a P-value cut-off of P < 0.05 were selected. Heatmaps of selections of enriched genes were generated with Gene-E ( http://www.broadinstitute.org/cancer/software/GENE-E/index.html). Genes were hierarchical clustered through the one minus Pearson correlation. In order to be able to predict temporal gene networks based on our selection of enriched genes in MESP1-positive derivatives, we screened genes on DNA binding domains and transcription factor activity (using Gene Set Enrichment Analysis (GSEA) at www.broadinstitute.org), and used the STRING database to integrate protein-protein interactions. This online available database provides a prediction pipeline for inferring protein-protein associations, covering more than 2000 organisms, with scalable algorithms for transferring interaction information between organisms, based on prediction, and *in vivo* and *in vitro* experimental assays, including gene co-occurrence in genomes (i.e. phylogeny), gene co-expression, gene fusion events, genomic neighbourhood (i.e. synteny), text mining, and experimental data such as co-immunoprecipitation and yeast two hybrid^6^. STRING extract experimental data from BIND, DIP, GRID, HPRD, IntAct, MINT, and PID databases. Cluster positions in the network are determined by an algorithm that is based on global confidence binding score (medium  > 0.4 or high > 0.7). Based on this score, clustering of gene nodes (visualized by node colours) was determined by applying the Markov Cluster Algorithm^53^. Thus, interacting proteins with a higher global score have more chance to end up in the same cluster.

In order to understand how developmental networks are build up throughout cardiac differentiation, we extracted STRING data into Cytoscape^54^ and generated a large interrogating network from protein-protein interactions from day 5, day 7, and day 10 (interactions with a high confidence >0.7 are visualized).

## Additional Information

**How to cite this article**: den Hartogh, S. C. *et al.* A comprehensive gene expression analysis at sequential stages of *in vitro* cardiac differentiation from isolated MESP1-expressing-mesoderm progenitors. *Sci. Rep.* **6**, 19386; doi: 10.1038/srep19386 (2016).

## Supplementary Material

Supplementary Information

## Figures and Tables

**Table 1:** **Gene Ontology Analysis of enriched transcripts at sequential stages of differentiation.**

Category	Term	Benjamini		
		Count	%	Corrected P-value
Day 5 (Enriched transcripts in M+ vs M−, FC > 1.5, P < 0.05)				
PANTHER_BP	BP00193:Developmental processes	89	20,60	1,35E-06
PANTHER_BP	BP00102:Signal transduction	108	25,00	0,011389832
PANTHER_BP	BP00274:Cell communication	47	10,88	0,018208241
PANTHER_BP	BP00248:Mesoderm development	27	6,25	0,020433632
PANTHER_BP	BP00246:Ectoderm development	31	7,18	0,016956308
PANTHER_BP	BP00199:Neurogenesis	27	6,25	0,032211571
PANTHER_MF	MF00178:Extracellular matrix	27	6,25	3,52E-05
PANTHER_MF	MF00179:Extracellular matrix structural protein	11	2,55	0,001599702
Day 7 (Enriched transcripts in M+ vs M−, FC > 1.5, P < 0.05)				
PANTHER_BP	BP00193:Developmental processes	38	24,36	0,002291487
PANTHER_BP	BP00124:Cell adhesion	16	10,26	0,015033261
PANTHER_BP	BP00281:Oncogenesis	12	7,69	0,059284076
PANTHER_BP	BP00274:Cell communication	22	14,10	0,046309515
PANTHER_BP	BP00102:Signal transduction	43	27,56	0,187888803
PANTHER_BP	BP00122:Ligand-mediated signaling	9	5,77	0,425197991
PANTHER_BP	BP00071:Proteolysis	15	9,62	0,405883178
PANTHER_BP	BP00109:Receptor protein serine/threonine kinase signaling pathway	3	1,92	0,531826987
PANTHER_BP	BP00248:Mesoderm development	10	6,41	0,535902255
PANTHER_BP	BP00103:Cell surface receptor mediated signal transduction	21	13,46	0,653573879
PANTHER_BP	BP00267:Homeostasis	5	3,21	0,667475861
PANTHER_MF	MF00178:Extracellular matrix	18	11,54	1,59E-06
PANTHER_MF	MF00282:Aspartic protease	4	2,56	0,035633978
PANTHER_MF	MF00179:Extracellular matrix structural protein	6	3,85	0,041276498
Day 10 (Enriched transcripts in M+ vs M−, FC > 1.5, P < 0.05)				
PANTHER_BP	BP00173:Muscle contraction	38	5,65	2,02E-14
PANTHER_BP	BP00250:Muscle development	25	3,71	9,55E-08
PANTHER_BP	BP00248:Mesoderm development	51	7,58	1,36E-06
PANTHER_BP	BP00193:Developmental processes	122	18,13	1,58E-04
PANTHER_BP	BP00251:Heart development	10	1,49	0,004440264
PANTHER_BP	BP00285:Cell structure and motility	65	9,66	0,019756301
PANTHER_BP_ALL	BP00287:Cell motility	27	4,01	0,018914324
PANTHER_MF	MF00261:Actin binding cytoskeletal protein	48	7,13	1,83E-11
PANTHER_MF	MF00091:Cytoskeletal protein	63	9,36	6,27E-06
PANTHER_MF	MF00178:Extracellular matrix	34	5,05	2,34E-04
PANTHER_MF	MF00230:Actin binding motor protein	9	1,34	0,024317475
Day 14 (Enriched transcripts in M+ vs M−, FC > 1.5, P < 0.05)				
PANTHER_BP	BP00173:Muscle contraction	35	0,41	1,00E-10
PANTHER_BP	BP00250:Muscle development	25	0,29	6,18E-07
PANTHER_BP	BP00248:Mesoderm development	55	0,64	6,46E-07
PANTHER_BP	BP00193:Developmental processes	141	1,65	9,67E-07
PANTHER_BP	BP00285:Cell structure and motility	80	0,94	8,87E-05
PANTHER_BP	BP00286:Cell structure	50	0,59	0,002674421
PANTHER_BP	BP00251:Heart development	10	0,12	0,006537938
PANTHER_BP	BP00287:Cell motility	30	0,35	0,006541566
PANTHER_BP	BP00109:Receptor protein serine/threonine kinase signaling pathway	8	0,09	0,032075749
PANTHER_MF	MF00261:Actin binding cytoskeletal protein	52	0,61	3,25E-12
PANTHER_MF	MF00091:Cytoskeletal protein	71	0,83	3,81E-07
PANTHER_MF	MF00178:Extracellular matrix	34	0,40	0,001924022
PANTHER_MF	MF00188:Select calcium binding protein	26	0,30	0,007733675
PANTHER_MF	MF00179:Extracellular matrix structural protein	13	0,15	0,008110282
PANTHER_MF	MF00230:Actin binding motor protein	9	0,11	0,031006045

Differentially expressed genes in the MESP1-mCherry positive derived cardiac populations were selected on a FC > 1.5 fold difference with their MESP1-mCherry negative counterpart, and a P-value < 0.05, n = 3. GO-analysis was performed using the Panther Classification System. Multiple-GO-term correction was performed using the Benjamini-Yekutieli method. GO-terms hold a corrected p-value < 0.05.

## References

[b1] Beqqali A., Kloots J., Ward-van Oostwaard D., Mummery C. & Passier R. Genome-Wide Transcriptional Profiling of Human Embryonic Stem Cells Differentiating to Cardiomyocytes. STEM CELLS 24, 1956–1967 (2006). 16675594 10.1634/stemcells.2006-0054

[b2] Paige S. L. *et al.* A Temporal Chromatin Signature in Human Embryonic Stem Cells Identifies Regulators of Cardiac Development. Cell 151, 221–232 (2012). 22981225 10.1016/j.cell.2012.08.027 PMC3462257

[b3] Elliott D. A. *et al.* NKX2-5eGFP/w hESCs for isolation of human cardiac progenitors and cardiomyocytes. Nat Meth 8, 1037–1040 (2011). 10.1038/nmeth.1740 22020065

[b4] Hartogh. Den S. C. *et al.* Dual Reporter MESP1 mCherry/w-NKX2-5 eGFP/whESCs Enable Studying Early Human Cardiac Differentiation. STEM CELLS 33, 56–67 (2014). 10.1002/stem.1842 25187301

[b5] Curtis R. K., Oresic M. & Vidal-Puig A. Pathways to the analysis of microarray data. Trends Biotechnol. 23, 429–435 (2005). 15950303 10.1016/j.tibtech.2005.05.011

[b6] Szklarczyk D. *et al.* STRING v10: protein-protein interaction networks, integrated over the tree of life. Nucleic Acids Research 43, D447–52 (2015). 25352553 10.1093/nar/gku1003 PMC4383874

[b7] Jacobs Y., Schnabel C. A. & Cleary M. L. Trimeric association of Hox and TALE homeodomain proteins mediates Hoxb2 hindbrain enhancer activity. Mol. Cell. Biol. 19, 5134–5142 (1999). 10373562 10.1128/mcb.19.7.5134 PMC84356

[b8] Chang C. P., Brocchieri L. & Shen W. F. Pbx modulation of Hox homeodomain amino-terminal arms establishes different DNA-binding specificities across the Hox locus. … and cellular biology (1996). 10.1128/mcb.16.4.1734 PMC231160 8657149

[b9] Phelan M. L., Rambaldi I. & Featherstone M. S. Cooperative interactions between HOX and PBX proteins mediated by a conserved peptide motif. Mol. Cell. Biol. 15, 3989–3997 (1995). 7623795 10.1128/mcb.15.8.3989 PMC230638

[b10] Stankunas K. *et al.* Pbx/Meis deficiencies demonstrate multigenetic origins of congenital heart disease. Circulation Research 103, 702–709 (2008). 18723445 10.1161/CIRCRESAHA.108.175489 PMC2633052

[b11] Laforest B., Bertrand N. & Zaffran S. Anterior Hox Genes in Cardiac Development and Great Artery Patterning. JCDD 1, 3–13 (2014).

[b12] Maves L. *et al.* Pbx homeodomain proteins direct Myod activity to promote fast-muscle differentiation. Development 134, 3371–3382 (2007). 17699609 10.1242/dev.003905

[b13] Berkes C. A. *et al.* Pbx marks genes for activation by MyoD indicating a role for a homeodomain protein in establishing myogenic potential. Mol. Cell 14, 465–477 (2004). 15149596 10.1016/s1097-2765(04)00260-6

[b14] Lindsley R. C., Gill J. G., Murphy T. L., Langer E. M. & Cai M. Mesp1 coordinately regulates cardiovascular fate restriction and epithelial-mesenchymal transition in differentiating ESCs. Cell Stem Cell 3, 55–68 (2008). 18593559 10.1016/j.stem.2008.04.004 PMC2497439

[b15] Cantor A. B. & Orkin S. H. Coregulation of GATA factors by the Friend of GATA (FOG) family of multitype zinc finger proteins. Semin. Cell Dev. Biol. 16, 117–128 (2005). 15659346 10.1016/j.semcdb.2004.10.006

[b16] Walton R. Z., Bruce A., Olivey H. E. & Najib K. Fog1 is required for cardiac looping in zebrafish. Developmental 289, 482–493 (2006). 10.1016/j.ydbio.2005.10.040 PMC2804444 16316643

[b17] Melnick A. *et al.* AML-1/ETO fusion protein is a dominant negative inhibitor of transcriptional repression by the promyelocytic leukemia zinc finger protein. Blood 96, 3939–3947 (2000). 11090081

[b18] Zhang L. *et al.* Characterization of a t(5;8)(q31;q21) translocation in a patient with mental retardation and congenital heart disease: implications for involvement of RUNX1T1 in human brain and heart development. Eur. J. Hum. Genet. 17, 1010–1018 (2009). 19172993 10.1038/ejhg.2008.269 PMC2986559

[b19] Huggins G. S., Bacani C. J., Boltax J., Aikawa R. & Leiden J. M. Friend of GATA 2 physically interacts with chicken ovalbumin upstream promoter-TF2 (COUP-TF2) and COUP-TF3 and represses COUP-TF2-dependent activation of the atrial natriuretic factor promoter. J. Biol. Chem. 276, 28029–28036 (2001). 11382775 10.1074/jbc.M103577200

[b20] Lu J. R. *et al.* FOG-2, a heart- and brain-enriched cofactor for GATA transcription factors. Mol. Cell. Biol. 19, 4495–4502 (1999). 10330188 10.1128/mcb.19.6.4495 PMC104407

[b21] Klinedinst S. L. & Bodmer R. Gata factor Pannier is required to establish competence for heart progenitor formation. Development 130, 3027–3038 (2003). 12756184 10.1242/dev.00517

[b22] Berry F. B., Miura Y., Mihara K., Kaspar P. & Sakata N. Positive and negative regulation of myogenic differentiation of C2C12 cells by isoforms of the multiple homeodomain zinc finger transcription factor ATBF1. Journal of Biological… 25057–25065 (2001). 10.1074/jbc.M010378200 11312261

[b23] Liu Y. *et al.* Genetic polymorphisms in ZFHX3 are associated with atrial fibrillation in a Chinese Han population. PLoS ONE 9, e101318 (2014). 24983873 10.1371/journal.pone.0101318 PMC4077770

[b24] Benjamin E. J. *et al.* Variants in ZFHX3 are associated with atrial fibrillation in individuals of European ancestry. Nat Genet 41, 879–881 (2009). 19597492 10.1038/ng.416 PMC2761746

[b25] Martin R. I. R. *et al.* Chromosome 16q22 variants in a region associated with cardiovascular phenotypes correlate with ZFHX3 expression in a transcript-specific manner. BMC Genet. 15, 136 (2014). 25539802 10.1186/s12863-014-0136-1 PMC4301889

[b26] Lian X. *et al.* Robust cardiomyocyte differentiation from human pluripotent stem cells via temporal modulation of canonical Wnt signaling(2). Proceedings of the… 109, 1848–1857 (2012). 10.1073/pnas.1200250109 PMC3390875 22645348

[b27] Witty A. D. *et al.* Generation of the epicardial lineage from human pluripotent stem cells. Nature Biotechnology 32, 1026–1035 (2014). 10.1038/nbt.3002 PMC4192149 25240927

[b28] Gessert S. & Kühl M. The multiple phases and faces of wnt signaling during cardiac differentiation and development. Circulation Research 107, 186–199 (2010). 20651295 10.1161/CIRCRESAHA.110.221531

[b29] Azhar M. *et al.* Transforming growth factor beta in cardiovascular development and function. Cytokine Growth Factor Rev. 14, 391–407 (2003). 12948523 10.1016/s1359-6101(03)00044-3 PMC3855389

[b30] Medici D., Hay E. D. & Goodenough D. A. Cooperation between snail and LEF-1 transcription factors is essential for TGF-beta1-induced epithelial-mesenchymal transition. Mol. Biol. Cell 17, 1871–1879 (2006). 16467384 10.1091/mbc.E05-08-0767 PMC1415320

[b31] Cheng P. *et al.* Fibronectin mediates mesendodermal cell fate decisions. Development 140, 2587–2596 (2013). 23715551 10.1242/dev.089052 PMC3666385

[b32] Lockhart M., Wirrig E., Phelps A. & Wessels A. Extracellular matrix and heart development. Birth Defects Research Part A: Clinical and Molecular Teratology 91, 535–550 (2011). 10.1002/bdra.20810 PMC3144859 21618406

[b33] Farouz Y., Chen Y., Terzic A. & Menasché P. Concise review: growing hearts in the right place: on the design of biomimetic materials for cardiac stem cell differentiation. STEM CELLS 33, 1021–1035 (2015). 25537366 10.1002/stem.1929

[b34] Turbendian H. K. *et al.* GATA factors efficiently direct cardiac fate from embryonic stem cells. Development 140, 1639–1644 (2013). 23487308 10.1242/dev.093260 PMC3621482

[b35] Palpant N. J., Pabon L., Rabinowitz J. S., Hadland B. K., Stoick-Cooper C. L., Paige S. L. *et al.* Transmembrane protein 88: a Wnt regulatory protein that specifies cardiomyocyte development. Development 140, 3799–3808 (2013). 23924634 10.1242/dev.094789 PMC3754478

[b36] Schoore G. V., Mendive F., Pochet R. & Vassart G. Expression pattern of the orphan receptor LGR4/GPR48 gene in the mouse. Histochem Cell Biol 124, 35–50 (2005). 16028069 10.1007/s00418-005-0002-3

[b37] Loh E. D., Broussard S. R. & Kolakowski L. F. Molecular characterization of a novel glycoprotein hormone G-protein-coupled receptor. Biochemical and Biophysical Research Communications 282, 757–764 (2001). 11401528 10.1006/bbrc.2001.4625

[b38] Evseenko D. *et al.* Mapping the first stages of mesoderm commitment during differentiation of human embryonic stem cells. Proceedings of the National Academy of Sciences 107, 13742–13747 (2010). 10.1073/pnas.1002077107 PMC2922221 20643952

[b39] Paxton C. N., Bleyl S. B., Chapman S. C. & Schoenwolf G. C. Identification of differentially expressed genes in early inner ear development. Gene Expr. Patterns 10, 31–43 (2010). 19913109 10.1016/j.gep.2009.11.002 PMC2818654

[b40] Descamps B. *et al.* Frizzled 4 regulates arterial network organization through noncanonical Wnt/planar cell polarity signaling. Circulation Research 110, 47–58 (2012). 22076635 10.1161/CIRCRESAHA.111.250936

[b41] Müller P.-S. *et al.* The fibronectin leucine-rich repeat transmembrane protein Flrt2 is required in the epicardium to promote heart morphogenesis. Development 138, 1297–1308 (2011). 21350012 10.1242/dev.059386 PMC3050662

[b42] Miller R. A., Christoforou N., Pevsner J., McCallion A. S. & Gearhart J. D. (2008). Efficient Array-Based Identification of Novel Cardiac Genes through Differentiation of Mouse ESCs. PLoS ONE . 3(5), e2176. 18478100 10.1371/journal.pone.0002176 PMC2364653

[b43] Palty R., Raveh A., Kaminsky I., Meller R. & Reuveny E. (2012). SARAF Inactivates the Store Operated Calcium Entry Machinery to Prevent Excess Calcium Refilling. Cell . 149(2), 425–438 (2012). 22464749 10.1016/j.cell.2012.01.055

[b44] Dubois N. C. *et al.* SIRPA is a specific cell-surface marker for isolating cardiomyocytes derived from human pluripotent stem cells. Nature Biotechnology 29, 1011–1018 (2011). 10.1038/nbt.2005 PMC4949030 22020386

[b45] Skelton R., Costa M., Anderson D. J. & Bruveris F. (2014). SIRPA, VCAM1 and CD34 identify discrete lineages during early human cardiovascular development. Stem Cell Research , 13, 172–179. 24968096 10.1016/j.scr.2014.04.016

[b46] Amigo J. D. *et al.* The role and regulation of friend of GATA-1 (FOG-1) during blood development in the zebrafish. Blood 114, 4654–4663 (2009). 19729519 10.1182/blood-2008-12-189910 PMC2780302

[b47] Orkin S. H. Diversification of haematopoietic stem cells to specific lineages. Nat Rev Genet 1, 57–64 (2000). 11262875 10.1038/35049577

[b48] Chan S. S.-K. *et al.* Mesp1 Patterns Mesoderm into Cardiac, Hematopoietic, or Skeletal Myogenic Progenitors in a Context-Dependent Manner. Cell Stem Cell 12, 587–601 (2013). 23642367 10.1016/j.stem.2013.03.004 PMC3646300

[b49] Saga Y. Mesp1 Expression Is the Earliest Sign of Cardiovascular Development. TCM 10, 345–352 (2001). 11369261 10.1016/s1050-1738(01)00069-x

[b50] Carmon K. S., Gong X., Lin Q., Thomas A. & Liu Q. R-spondins function as ligands of the orphan receptors LGR4 and LGR5 to regulate Wnt/β-catenin signaling. PNAS 108, 11452–11457 (2011). 21693646 10.1073/pnas.1106083108 PMC3136304

[b51] Yi J. *et al.* Analysis of LGR4 receptor distribution in human and mouse tissues. PLoS ONE 8, e78144 (2013). 24205130 10.1371/journal.pone.0078144 PMC3804454

[b52] Barker N., Tan S. & Clevers H. Lgr proteins in epithelial stem cell biology. Development 140, 2484–2494 (2013). 23715542 10.1242/dev.083113

[b53] Brohée S. & van Helden J. Evaluation of clustering algorithms for protein-protein interaction networks. BMC Bioinformatics 7, 488 (2006). 17087821 10.1186/1471-2105-7-488 PMC1637120

[b54] Shannon P. *et al.* Cytoscape: a software environment for integrated models of biomolecular interaction networks. Genome Res. 13, 2498–2504 (2003). 14597658 10.1101/gr.1239303 PMC403769

