# Supplementary material for: A comprehensive gene expression analysis at sequential stages of in vitro cardiac differentiation from isolated MESP1-expressing-mesoderm progenitors
Source: Sci Rep. 2016 Jan 19;6:19386. doi: 10.1038/srep19386 (PMC4726039; doi:10.1038/srep19386)
Supplement: Supplementary Information [file srep19386-s1.pdf]

A comprehensive gene expression analysis at sequential stages of *in vitro* cardiac differentiation from isolated MESP1-expressing-mesoderm progenitors.

Sabine C. den Hartogh[1], Katherine Wolstencroft[2], Christine L. Mummery[1], Robert Passier[1,3*].

[1] Department of Anatomy and Embryology, Leiden University Medical Centre, Leiden, The Netherlands.

[2] Leiden Institute of Advanced Computer Science Leiden Institute of Advanced Computer Science, Leiden University, The Netherlands.

[3] Department of Applied Stem cell Technologies. MIRA Institute for Biomedical Technology and Technical Medicine. University of Twente, P.O.Box 217, Enschede, The Netherlands.

*** Corresponding author:** robert.passier@utwente.nl.

Supplementary Information

Figure S1. Heatmaps visualize the relative expression levels of transcripts from other lineages, throughout cardiac differentiation. Smooth muscle cells and endothelial (progenitor) cells are enriched in MESP1-mCherry positive derivatives. Endoderm- and skeletal- related transcripts show no enrichment in the MESP1-positive derivatives. Only a selected number of hematopoietic-lineage-related transcripts, including CD34, HBE1 and HBG1 show an increase, indicating the sparse numbers of MESP1-derived hematopoietic lineages in our cultures. Heatmaps show averaged values from n=3.

Table S1a. Top 100 lists of enriched transcripts, ordered by Fold Change.

Table S1b. Top 100 lists of downregulated transcripts, ordered by Fold Change.

Table S2. KEGG Pathway Analysis of enriched transcripts in MESP1+ derivatives versus MESP1- derivatives (P<0.05, FC>1.5, n=3). Pathways are selected based on p-value < 0.05.

Supplementary Figure 1. Other lineages

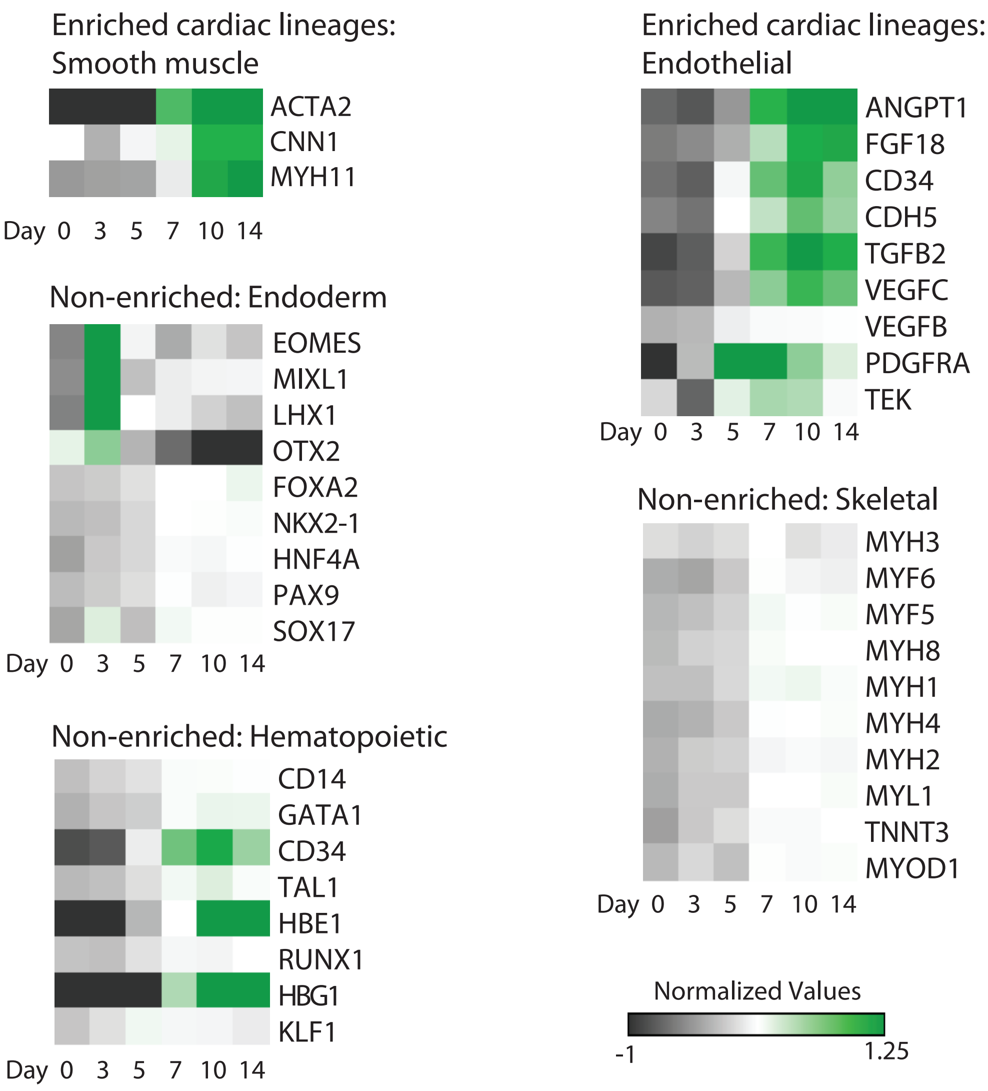

Supplemental Table 1a. Top 100 lists of enriched transcripts, ordered by Fold Change.

Day 5: M+X+ vs M-X+, FC>1.5, P<0.05

				Normalized values:	
**Symbol**	**FC (abs)**	**Corrected p-value**	**P-value**	**[D5 M+]**	**[D5 M-]**
PANX2	11,259163	4,10E-09	2,82E-12	3,55569	0,062662445
CALB2	7,6899123	2,17E-07	6,24E-10	3,4189415	0,4759744
LOC100134265	6,442907	9,56E-07	4,18E-09	3,0187886	0,33107695
COL22A1	5,591488	2,02E-06	1,13E-08	0,073375225	-2,409857
LOC728473	5,3762	1,22E-06	5,72E-09	2,00082	-0,42576694
SMOC1	4,8788123	5,53E-08	9,11E-11	0,73825663	-0,34929833
ATP6V1B1	4,4719763	4,66E-07	1,65E-09	1,6459465	-0,514966
SFRP5	4,171084	6,78E-05	8,83E-07	3,4223273	1,361905
LOC100128893	4,012275	2,69E-06	1,59E-08	1,1306558	-0,36707845
PTGIS	3,9306362	4,87E-07	1,78E-09	2,2842686	0,3095058
PPP1R1A	3,783339	7,15E-05	9,46E-07	1,6093072	-0,31035295
PTGDS	3,7469463	1,08E-07	2,52E-10	1,7762278	-0,12948751
CD82	3,5785627	3,60E-05	4,09E-07	1,1695186	-9,82E-04
COL2A1	3,4955988	1,11E-05	9,10E-08	1,5433607	-0,2621789
NR0B1	3,4516559	2,55E-06	1,48E-08	1,6549202	-0,1323684
MEIS2	3,3875015	1,04E-06	4,63E-09	1,341197	-0,41902462
FLRT2	3,359133	2,12E-05	2,09E-07	1,5133996	-0,2346894
OSR1	3,3569884	2,02E-06	1,14E-08	1,3493775	-0,3977901
SC5DL	3,3354106	1,63E-06	8,37E-09	1,9730573	0,23519294
RBM20	3,3253138	3,33E-07	1,10E-09	1,4832035	-0,2502869
FAM46A	3,309693	1,66E-05	1,52E-07	1,271986	-0,45471144
AKR1C3	3,2239087	0,003333452	1,32E-04	2,2299836	0,5411728
FGFRL1	3,2019522	1,56E-05	1,40E-07	1,8669511	0,18799941
FNDC5	3,192409	4,79E-07	1,73E-09	1,7400657	0,06542015
MYL7	3,1884224	2,17E-05	2,17E-07	0,72645855	-0,94638425
ZNF503	3,1578243	3,30E-04	6,95E-06	1,5776267	-0,08130423
FZD4	3,138556	1,99E-07	5,43E-10	2,0260437	0,3759427
AKR1C2	3,073294	5,13E-04	1,21E-05	1,0958034	-0,52398235
RARB	3,028789	1,16E-05	9,61E-08	0,9072134	-0,11634954
PGA5	2,9732735	1,51E-04	2,54E-06	3,4088833	1,8368311
PDGFRA	2,9545476	1,30E-06	6,19E-09	1,8832884	0,32825232
WDR86	2,939531	6,33E-06	4,53E-08	1,4872271	-0,0683589
NFKBIZ	2,9277465	1,13E-07	2,71E-10	1,4186484	-0,1311423
LAMA1	2,8969975	9,65E-08	2,10E-10	1,846944	0,31238556
CCKBR	2,8821356	1,41E-04	2,28E-06	1,8074466	0,2803084
HBE1	2,8791997	0,001062561	2,96E-05	-0,6262212	-2,151889
IRX5	2,8677714	7,08E-05	9,32E-07	1,3617998	-0,15813017
PGA3	2,8404906	1,87E-04	3,35E-06	3,1320155	1,6289424
IGF2	2,8376665	2,12E-06	1,20E-08	2,7573872	1,2598726
MEIS1	2,8360891	4,79E-07	1,73E-09	1,6443901	0,1404872
CDC42EP5	2,7935421	5,30E-06	3,71E-08	1,3777179	-0,10437775

HAPLN3	2,7358084	3,10E-05	3,36E-07	1,6540936	0,2021265
LAMA1	2,7108698	6,80E-06	5,01E-08	1,6627396	0,22398376
KCNA5	2,7104564	6,27E-04	1,55E-05	0,9107941	-0,52774173
PLCE1	2,6811774	1,30E-06	6,14E-09	1,8428359	0,41996923
HOXB2	2,6652071	1,04E-06	4,69E-09	1,4034468	-0,010800839
PGF	2,6470728	6,92E-05	9,08E-07	1,6074662	0,20306842
ENO3	2,6467905	9,27E-06	7,19E-08	0,24326913	-0,90233725
DACT1	2,636526	7,19E-05	9,64E-07	0,66285783	-0,7357804
FER1L3	2,6102366	1,16E-04	1,76E-06	0,4155469	-0,63862705
LIX1	2,5894253	1,01E-05	8,00E-08	1,9429487	0,5703168
STC2	2,5849762	2,91E-04	5,90E-06	0,9344533	-0,4356977
METRN	2,5588253	1,82E-05	1,71E-07	1,5876607	0,232179
N4BP2L1	2,550709	2,72E-04	5,36E-06	1,2314934	0,057509262
RBP1	2,5154676	7,84E-06	5,98E-08	1,3382668	0,007440249
DNAH2	2,4896827	9,44E-07	4,10E-09	2,111231	0,7952693
PBX3	2,4819348	1,67E-05	1,53E-07	2,0584934	0,74702805
IRX3	2,4738026	5,30E-06	3,69E-08	1,6235743	0,31684384
SCMH1	2,4683418	3,44E-05	3,82E-07	0,62616986	-0,12673204
C15orf39	2,4574416	3,23E-06	2,01E-08	1,3388128	0,0416557
NAV1	2,4539363	2,46E-06	1,41E-08	0,62848634	-0,6666115
DUSP5	2,4120514	3,45E-04	7,40E-06	1,2260895	-0,044171173
KCNAB1	2,4063036	3,61E-04	7,84E-06	1,6281029	0,36128426
PRRX2	2,3957248	3,13E-06	1,93E-08	2,365259	1,1047968
ATF7IP2	2,3807654	4,79E-07	1,71E-09	1,4664856	0,21506007
TIPARP	2,3455749	1,81E-05	1,69E-07	0,7446688	-0,48527274
AKR1C4	2,3369477	0,006375599	3,04E-04	0,83792686	-0,38669857
AKR1B15	2,314229	0,001096666	3,10E-05	0,9591935	-0,25133815
FOXF1	2,3105698	2,14E-05	2,14E-07	1,2030977	-0,005150954
COLEC12	2,3092504	6,54E-06	4,80E-08	1,6569368	0,44951215
NAT8B	2,30729	5,97E-05	7,56E-07	0,93336123	-0,27283812
ALDH1A2	2,2865431	0,041177012	0,003872786	1,5179497	0,32478157
B3GALNT1	2,270695	7,12E-06	5,31E-08	0,8105051	-0,37262884
SLC16A3	2,2623792	0,005917038	2,75E-04	0,612669	-0,5651717
NPY	2,2619336	0,001516515	4,77E-05	1,1821562	0,00459973
COL11A1	2,2424514	6,80E-05	8,87E-07	0,85233384	-0,1634183
ODAM	2,2421424	4,73E-05	5,70E-07	1,2537984	0,08892044
SLC17A8	2,2386634	0,007560806	3,80E-04	0,96278477	-0,19985278
C8orf13	2,2364564	1,86E-05	1,78E-07	0,71952325	-0,4416914
ARHGAP28	2,2324505	2,09E-04	3,84E-06	0,8648222	-0,03041776
COL9A2	2,2250178	2,71E-04	5,32E-06	2,1273384	0,9735214
ST3GAL5	2,2247953	5,30E-06	3,71E-08	1,5472322	0,39355946
MYOF	2,2167444	2,85E-04	5,74E-06	0,45094156	-0,697501
CTSB	2,2030556	6,78E-05	8,82E-07	0,48840857	-0,6510973
CXCR7	2,192779	7,88E-04	2,04E-05	0,07674185	-1,0560187
CPE	2,1917334	4,04E-04	8,97E-06	0,3340257	-0,7980466
TMEM88	2,1897798	7,19E-05	9,67E-07	2,8199694	1,6891837

REC8	2,182012	7,24E-06	5,44E-08	1,2151872	0,3210945
FAM167A	2,16832	3,48E-05	3,92E-07	0,66311675	-0,45346084
WFIKKN1	2,1643171	6,18E-05	7,88E-07	1,2357521	0,12184016
MMP11	2,1607587	0,009259991	4,97E-04	1,015639	-0,095899105
LRRTM1	2,1604922	0,001290751	3,89E-05	1,7659067	0,65454656
USP24	2,15969	1,13E-04	1,70E-06	1,443349	0,33252493
TMTC1	2,157107	1,61E-04	2,77E-06	1,1458038	0,03670597
CNTFR	2,155216	1,59E-04	2,73E-06	0,8680795	-0,23975293
IRX5	2,1513429	1,43E-04	2,33E-06	0,78451014	-0,32072735
H2AFY	2,1500185	1,28E-04	2,01E-06	0,9796397	0,12656863
CYB5D1	2,1421564	0,002038979	6,99E-05	2,1968417	1,097778
FAM38A	2,1275485	3,20E-04	6,63E-06	1,3143731	0,22518127
COL2A1	2,1237342	0,001340591	4,07E-05	0,99571943	-0,09088389

Day 7: M+X+ vs M-X+, FC>1.5, P<0.05

				Normalized values:	
**Symbol**	**FC (abs)**	**Corrected p-value**	**P-value**	**[D7 M+]**	**[D7 M-]**
COL11A1	3,9346414	2,64E-05	3,97E-09	1,1718909	-0,019676208
NR2F1	3,7769258	4,69E-05	1,41E-08	1,1156273	-0,8015852
SRD5A2L2	3,7583373	1,37E-04	6,97E-08	2,169092	0,25899744
C20orf103	3,7308252	4,13E-04	5,90E-07	1,7007076	-0,19878721
CALB2	3,0993328	5,52E-05	2,13E-08	1,7860432	0,15408547
LOC728473	3,0684054	0,002631484	1,37E-05	1,5485471	-0,06894191
IGFBP5	2,9625895	3,40E-04	3,96E-07	-0,09096146	-1,6081824
PGA5	2,9036307	0,030666973	5,75E-04	2,21126	0,673402
PDGFRA	2,840711	8,53E-04	2,31E-06	1,4483479	0,14898904
FLRT2	2,8228939	0,001018372	3,17E-06	1,0981289	-0,39904594
AMHR2	2,7762063	3,40E-04	4,07E-07	1,672362	0,1992472
RBP1	2,7497501	5,98E-04	1,23E-06	1,0058349	-0,4534656
SFRP5	2,7469423	5,10E-04	9,06E-07	1,5706531	0,112826504
LIX1	2,7378254	0,005244703	3,70E-05	1,4535661	5,36E-04
TXNIP	2,7351766	0,0480669	0,001194227	0,23410685	-1,217527
LHX2	2,7288413	0,031791985	6,19E-04	0,651882	-0,79640645
PGA3	2,6921554	0,032383993	6,33E-04	1,401213	0,29800463
LOC100128893	2,6734674	0,011423129	1,21E-04	0,7810259	-0,04814895
CPE	2,6626234	2,38E-04	1,93E-07	1,4817443	0,06889597
REN	2,6403496	0,004810309	3,18E-05	1,9780264	0,5772975
DNAH2	2,6293375	0,014088605	1,70E-04	2,005183	0,61048365
RASD1	2,544676	0,026647715	4,48E-04	1,0732256	-0,27425638
ZNF503	2,5232937	0,001611862	6,17E-06	1,4187555	0,0834473
FZD4	2,4952683	0,002757089	1,49E-05	1,3328404	0,01364549
HAND2	2,4878957	0,005733177	4,34E-05	1,6861477	0,3712217
COL22A1	2,4442146	0,042371936	9,68E-04	1,6649755	0,37560448
SHISA2	2,4410539	0,008215486	7,16E-05	1,2317147	-0,05578931
GUCY1A3	2,3906515	3,61E-04	4,43E-07	0,8958616	0,02636846
PANX2	2,3675423	0,00250654	1,25E-05	1,6277828	0,3843926

GLT25D2	2,3042169	0,010749198	1,09E-04	1,3346447	0,13036823
RDH10	2,2699432	0,011000386	1,15E-04	0,7994483	-0,38320795
LRRTM1	2,2528954	0,004805083	3,16E-05	1,2128243	0,041044075
SLIT2	2,2312794	2,35E-04	1,83E-07	1,3467474	0,18887632
LOC100134265	2,2165396	2,59E-04	2,47E-07	1,2538885	0,105579376
SLC30A3	2,2082758	0,004891709	3,27E-05	1,5495296	0,4066092
SULF2	2,1918497	0,014893705	1,85E-04	0,8112095	-0,3209394
DHRS9	2,1794584	0,03079564	5,80E-04	1,6488796	0,52491
SVEP1	2,1699255	0,009606232	9,06E-05	1,2030934	0,085447945
SFRP1	2,1518848	6,60E-04	1,45E-06	0,5265514	-0,5790494
RNF150	2,1428473	0,005733177	4,31E-05	1,186845	0,08731588
PTPRD	2,1359088	2,02E-04	1,37E-07	1,0320135	-0,06283649
RELN	2,1205986	0,03900831	8,43E-04	1,8176364	0,7331648
MEIS2	2,0926356	0,015705014	2,01E-04	1,1347684	0,0694472
KEL	2,068549	6,57E-04	1,42E-06	1,3666106	0,31799158
COL6A2	2,038893	0,02713714	4,59E-04	0,7851098	-0,24267626
OSAP	2,0192	0,03254569	6,38E-04	1,1028117	0,08902788
EFNB3	2,0081832	0,002585767	1,33E-05	0,9512181	-0,05467288
LRRN3	1,9778239	7,65E-04	1,91E-06	0,62976074	0,017863592
C4orf49	1,9672202	0,04219164	9,60E-04	1,0738001	0,097641625
FAM46A	1,9642816	0,005733177	4,34E-05	0,79062957	-0,18337218
WNT5A	1,9623226	0,009107764	8,32E-05	0,6895587	-0,28300348
COL1A1	1,9531417	0,024180822	3,87E-04	0,9744622	0,008665562
LRRC32	1,950626	0,011465372	1,22E-04	1,0377842	0,07384697
LRRC3B	1,9491494	0,03874952	8,29E-04	0,8935192	-0,06932545
PTN	1,947436	0,00972477	9,26E-05	0,63678616	-0,32478967
ODC1	1,9474353	0,0079702	6,82E-05	0,06496032	-0,896615
LRCH2	1,9440354	0,001589488	5,92E-06	1,1324086	0,17335415
BMP5	1,9419221	0,001783626	7,21E-06	1,228672	0,27118668
CST3	1,9345984	0,005766487	4,40E-05	0,4876213	-0,46441284
CCRK	1,9314449	0,002662404	1,41E-05	0,55844945	-0,06254864
SNCAIP	1,930095	0,002725445	1,46E-05	0,8499519	-0,09871992
PBX3	1,904425	0,001413572	4,98E-06	0,9226672	-0,006688277
NAALAD2	1,895423	0,002047086	8,95E-06	1,0975188	0,17499892
PCOLCE	1,8853873	0,006429103	5,16E-05	0,95395964	0,03909874
CNTNAP4	1,8836802	0,002452352	1,20E-05	1,1899918	0,27643776
FNDC5	1,8653816	0,00326204	1,82E-05	0,94054383	0,041073006
SPON1	1,8618454	0,03807662	8,04E-04	0,8525386	-0,0441947
SHROOM3	1,8491266	0,04551982	0,00110233	0,7947734	-0,09207058
CDC42EP5	1,8443766	0,031484112	6,01E-04	0,78734016	-0,09579309
SMOC1	1,8389484	0,034486145	6,98E-04	0,77683544	-0,102045536
ZFPM2	1,8378743	0,035348207	7,22E-04	0,82491046	-0,053127605
STRA6	1,8313638	0,018945696	2,68E-04	1,0502424	0,17732398
LAMA1	1,8233843	0,002008862	8,54E-06	0,9322913	0,18311627
TSHZ2	1,821756	0,017277937	2,31E-04	0,78417903	-0,08115069
PCDH17	1,8101013	0,012664752	1,45E-04	0,709861	-0,1462094

TIPARP	1,8092344	0,006212326	4,91E-05	0,30648994	-0,54888934
NCCRP1	1,8082901	0,045942206	0,00112081	1,0276203	0,17299414
NFIB	1,8076339	0,012182911	1,37E-04	0,3907671	-0,46333537
RGL1	1,8063636	0,00529416	3,79E-05	0,74227875	-0,11080948
ST8SIA1	1,8021686	0,002585767	1,32E-05	0,8538324	0,004098415
COL5A2	1,7995836	0,025399758	4,15E-04	0,47147384	-0,37618923
CSRP2	1,7933767	0,023618635	3,75E-04	0,62602156	-0,216657
PLCE1	1,7929716	0,004206932	2,56E-05	0,8449497	0,002597014
IMPAD1	1,7929487	8,53E-04	2,33E-06	0,72873545	-0,11359882
LBA1	1,7827694	0,010482166	1,05E-04	0,8655095	0,031389397
LOC131873	1,7808284	0,013013354	1,52E-04	0,8219139	-0,010634582
CD34	1,7708908	0,018015042	2,49E-04	0,7961545	-0,017919382
CCDC3	1,7652751	0,01248897	1,42E-04	0,5573804	-0,26251268
MDK	1,7614261	0,016884444	2,24E-04	0,7318552	-0,08488878
ATP1B1	1,7597347	0,027845116	4,75E-04	0,78445417	0,027939161
AEBP1	1,7547044	0,011634754	1,25E-04	0,58750504	-0,22372293
KLF11	1,7541358	0,0367682	7,62E-04	0,420369	-0,3903915
COL8A1	1,7429901	0,007555558	6,40E-05	0,63361377	-0,16795063
EPB41L3	1,740704	0,004805083	3,16E-05	0,79179984	-0,007871151
CTSB	1,7382442	0,039393436	8,62E-04	0,66936094	-0,100790024
CD99L2	1,718871	0,002279656	1,08E-05	0,5499752	-0,23148601
AXL	1,7092984	0,0350756	7,14E-04	0,573637	-0,19976728
COL6A6	1,7036593	0,01869213	2,62E-04	0,7791791	0,010542234
SMAD3	1,6952828	0,01385025	1,66E-04	0,5490532	-0,21247275
RUNX1T1	1,6949229	0,025595598	4,22E-04	0,7564618	-0,004757881

Day 10: M+X+ vs M-X+, FC>1.5, P<0.05

				Normalized values:	
**Symbol**	**FC (abs)**	**Corrected p-value**	**P-value**	**[D10 M+]**	**[D10 M-]**
PGA5	4,990784	2,42E-07	3,40E-10	2,1691258	-0,15014076
PGA3	4,783451	3,21E-07	4,92E-10	1,4337467	-0,031412285
TNNT2	4,474228	5,53E-05	5,10E-07	2,9339073	1,5281216
RASD1	4,2666326	1,33E-07	1,55E-10	1,678885	-0,41421285
AMHR2	4,2563596	3,14E-08	1,97E-11	1,7916045	-0,2980156
SYNPO2L	4,1732197	4,25E-06	1,58E-08	2,8318775	0,77071667
FIT1	4,1247497	4,24E-06	1,55E-08	2,9364102	0,8921037
MYL3	3,9280941	3,96E-04	6,93E-06	4,686685	2,7128556
CRIP2	3,8027978	6,97E-05	6,91E-07	1,9796158	0,052554607
GATM	3,7152016	7,93E-07	1,58E-09	2,2037194	0,3102789
SORBS2	3,678383	1,29E-05	7,38E-08	2,418882	0,8274167
SRD5A2L2	3,6308212	1,21E-06	3,01E-09	2,299493	0,4391972
COL8A1	3,5548835	3,04E-06	9,47E-09	0,5966632	-0,04447158
SLC30A3	3,5505064	1,90E-07	2,33E-10	1,5986195	-0,22940524
HRC	3,5302114	1,55E-05	9,77E-08	2,9986954	1,1789408
MYOM1	3,5227978	4,02E-04	7,08E-06	4,071885	2,2551634
ADPRHL1	3,4543672	1,01E-04	1,16E-06	1,1314297	0,31850448

DHRS9	3,4175363	4,59E-06	1,76E-08	1,3074266	0,24616988
COL22A1	3,4160268	1,15E-05	6,38E-08	1,9797403	0,20742099
HAND2	3,3981016	4,03E-08	2,77E-11	1,8021194	0,037390392
FLJ14712	3,3825989	2,12E-07	2,84E-10	1,6881719	-0,069960274
TNNC1	3,3583596	5,01E-04	9,31E-06	3,8665574	2,1188006
HSPB7	3,3386488	1,92E-04	2,66E-06	3,0820303	1,3427659
MYBPC3	3,3260617	0,001728668	4,75E-05	3,7393312	2,0055163
ENO3	3,303148	2,66E-04	4,08E-06	1,7903951	0,20128362
LOC728473	3,2792938	4,15E-07	7,20E-10	1,2579465	-0,4554386
WNT2	3,2632136	2,63E-07	3,86E-10	1,9132657	0,20697229
MB	3,2361982	0,002786809	9,01E-05	2,784674	1,303636
ANGPT1	3,233352	7,04E-05	7,02E-07	1,811017	0,51438254
SMPX	3,2082663	6,99E-04	1,47E-05	2,0190003	0,6763379
EEF1A2	3,1733875	0,002402265	7,34E-05	1,360042	-0,30598179
NKX2-5	3,1653342	1,01E-05	5,16E-08	2,466832	0,80447406
KLF2	3,1530042	3,17E-08	2,09E-11	1,3730167	-0,28371033
IRX5	3,1411262	1,02E-05	5,28E-08	1,089179	-0,014375846
SRL	3,1362321	0,001649333	4,47E-05	2,9062855	1,2572532
RBM20	3,1287036	1,09E-06	2,57E-09	1,4868063	-0,1587588
HSPB3	3,1203425	0,002673007	8,49E-05	3,0012677	1,3595632
TCEA3	3,1199584	0,001270291	3,17E-05	2,7126243	1,0710975
FZD4	3,1180682	5,72E-07	1,04E-09	0,58487606	-1,0557765
PPP1R3C	3,1150582	5,83E-06	2,39E-08	1,3150522	-0,32420698
SLIT2	3,0889585	5,08E-05	4,40E-07	1,4757212	-0,1513993
TMOD1	3,0845537	3,91E-04	6,74E-06	2,445983	0,8209211
SMYD1	3,0668156	1,01E-04	1,15E-06	2,97239	1,3556485
PLAT	3,0543153	7,80E-04	1,70E-05	1,24295	-0,3678991
SPHKAP	3,0524108	3,83E-06	1,33E-08	2,1823452	0,5723961
FBXO32	3,0391095	8,57E-07	1,86E-09	1,0859265	0,043314617
CKM	3,008675	0,005199002	2,00E-04	3,4639246	1,8747963
PLN	3,001282	3,76E-04	6,45E-06	2,4323437	0,84676486
MASP1	2,9897869	0,001071984	2,56E-05	1,4968162	0,40927902
GUCY1A3	2,989088	3,28E-06	1,05E-08	0,35418096	-0,2367425
GATA5	2,9870496	3,09E-07	4,62E-10	1,1930257	-0,38569546
RBM24	2,976125	2,21E-06	6,61E-09	1,3938531	-0,17958196
RFTN1	2,9683454	5,71E-07	1,03E-09	0,9081793	-0,6614798
ACTA2	2,925816	1,78E-06	4,90E-09	1,9386206	0,38978148
PTPLA	2,8755987	3,31E-04	5,44E-06	1,4117109	-0,11215147
MEF2C	2,8660915	9,23E-06	4,56E-08	1,7035736	0,18448894
PDGFRA	2,8603485	9,10E-04	2,06E-05	0,7033129	-0,6523827
TSPAN32	2,8364859	8,82E-05	9,85E-07	1,3001881	0,32879066
APOBEC2	2,8328414	0,006130025	2,46E-04	2,8996124	1,3973626
C11orf9	2,831818	4,34E-04	7,79E-06	1,4880334	-0,01369524
MATN2	2,821055	8,57E-07	1,87E-09	0,8891926	0,11283287
MYH7	2,8063917	0,042632896	0,003038553	3,2627761	1,7740598
LOC441081	2,803444	0,003492705	1,22E-04	2,3005369	0,81333655

ATP2A2	2,7726607	1,36E-06	3,51E-09	1,8207369	0,48390308
DPP7	2,7701905	3,36E-05	2,53E-07	0,7166807	-0,05497217
LDB3	2,7639527	1,42E-04	1,82E-06	2,2797632	0,81303024
LBH	2,7629404	2,63E-07	3,77E-10	0,77748746	0,0571812
BMP5	2,7494707	3,84E-07	6,55E-10	1,4600803	9,26E-04
RBP1	2,7479367	2,12E-07	2,74E-10	0,80909204	-0,6492567
GJA3	2,739333	1,45E-05	8,88E-08	-1,1089934	-0,47407055
SLC25A4	2,724574	1,19E-05	6,71E-08	1,1060375	-0,067286015
SEPT5	2,7068026	0,002248355	6,74E-05	2,3507984	0,9142087
KCNA5	2,6966243	2,50E-05	1,78E-07	1,339738	-0,091416515
PPP1R13B	2,696039	8,51E-07	1,78E-09	1,8227128	0,39187145
LOC100128893	2,6941392	3,93E-05	3,06E-07	1,0947104	-0,33511415
ACTN2	2,6819568	1,44E-05	8,76E-08	2,2437787	0,82049274
PPP1R1A	2,6671371	4,25E-06	1,58E-08	1,5469369	0,13164489
NDRG2	2,6652007	1,02E-05	5,29E-08	1,2412621	0,026466688
CRIP1	2,6571941	0,002741904	8,79E-05	1,6864401	0,27653646
SLC47A1	2,6571298	1,37E-05	8,14E-08	1,179524	-0,23034477
SH3RF2	2,6455696	0,001601614	4,31E-05	1,3687983	-0,034780186
PRICKLE1	2,6391609	1,00E-06	2,31E-09	1,0519639	-0,3481153
TNNI3	2,609821	5,44E-04	1,04E-05	1,936338	0,55238694
C9orf61	2,6003323	1,29E-04	1,61E-06	1,8292607	0,4505647
MYL7	2,5989785	7,60E-04	1,64E-05	1,701636	0,32369122
SST	2,5962229	2,30E-05	1,60E-07	1,5936724	0,21725814
NEXN	2,5929644	2,48E-06	7,51E-09	1,3415627	-0,03303973
PDLIM3	2,5747838	4,01E-06	1,40E-08	0,9045882	-0,15451717
NFIB	2,5712173	3,81E-05	2,94E-07	1,1362723	-0,22617929
MYL4	2,5679767	0,004156955	1,50E-04	2,540259	1,1796268
MAP1LC3A	2,552861	2,18E-05	1,49E-07	1,0029525	-0,34916243
CRYAB	2,5481877	5,01E-04	9,31E-06	2,1326277	0,7831562
PPP1R14C	2,5459702	0,0019832	5,73E-05	2,1544783	0,8062628
PPARGC1A	2,529353	7,02E-06	3,29E-08	1,3798542	0,04108588
LOC399959	2,520519	3,50E-06	1,16E-08	1,3897133	0,055992443
VCAM1	2,5144067	0,001642888	4,44E-05	2,046314	0,7160959
SFRP5	2,5112913	0,001247955	3,10E-05	1,19946	-0,12896936
NCCRP1	2,5060549	0,001896332	5,42E-05	0,9888794	0,11418263
MYL9	2,5027378	6,65E-06	2,98E-08	1,3523303	0,066211544
TGFB2	2,5018277	1,19E-06	2,87E-09	1,452654	0,12967157

Day 14: M+X+ vs M-X+, FC>1.5, P<0.05

				Normalized values:	
**Symbol**	**FC (abs)**	**Corrected p-value**	**P-value**	**[D14 M+]**	**[D14 M-]**
ITLN1	14,244069	0,002197182	4,62E-05	4,316649	0,4843596
MYBPC3	6,327399	0,002452805	5,40E-05	4,0940976	1,4324852
SYNPO2L	5,882358	0,001237455	1,90E-05	3,4919002	0,93550557
TNNC1	5,8000855	0,004027108	1,16E-04	3,9847307	1,4486564
MYH7	5,621656	0,005581834	1,87E-04	5,433235	2,94224

HSPB7	5,1011415	0,00277426	6,57E-05	3,516229	1,1654087
DHRS9	5,0237203	6,58E-05	1,65E-07	2,4509974	0,12224134
FIT1	5,003747	3,82E-04	3,02E-06	3,0451329	0,7221241
MB	4,939631	0,003245323	8,41E-05	3,8144982	1,5100948
MYOM1	4,9082375	0,006589661	2,41E-04	4,197958	1,9027528
EEF1A2	4,9007397	0,005668292	1,93E-04	1,5854044	-0,70759517
MYL3	4,868687	0,007462195	2,91E-04	4,8198724	2,5363395
LOC728473	4,7678213	2,99E-04	1,94E-06	1,257538	-0,9957922
AMHR2	4,761907	1,83E-04	8,82E-07	1,55372	-0,69781953
HSPB3	4,743651	0,007423305	2,88E-04	4,0327206	1,7867225
SPHKAP	4,556213	0,002833824	6,83E-05	3,532872	1,3450369
LBH	4,436668	1,09E-04	3,39E-07	1,4707943	-0,6786823
MYL2	4,2927284	0,002516858	5,67E-05	3,3330708	1,2311759
ADPRHL1	4,1914253	0,004740571	1,49E-04	2,9828424	0,91540146
MYL4	4,1086597	0,029003648	0,002008125	2,532761	0,4940931
SH3RF2	4,0439095	0,005100468	1,66E-04	1,7055969	-0,31015363
SRL	4,0356545	0,015282005	8,24E-04	3,0861683	1,0733656
TNNT2	4,020482	0,015650533	8,51E-04	2,9633243	0,9559557
TMOD1	3,9864724	0,00277426	6,59E-05	2,6905422	0,6954296
PRSS35	3,9137669	6,65E-04	7,37E-06	2,7749064	0,8063486
NFIB	3,8617208	1,20E-05	1,36E-08	1,8335875	-0,115656376
ATP2A2	3,8594503	3,22E-04	2,19E-06	1,5539652	-0,39443016
SMYD1	3,8378944	0,013292648	6,76E-04	2,850703	0,910388
RYR2	3,8120332	3,36E-04	2,38E-06	1,9970632	0,066502415
MYL9	3,799799	1,54E-04	6,34E-07	1,732155	-0,19376819
HRC	3,7735755	0,006758382	2,48E-04	2,7491398	0,8332076
ALDH1A2	3,7521586	0,002114064	4,33E-05	1,298611	-0,6091097
TCEA3	3,6954134	0,019093333	0,001120784	2,933244	1,0475082
RPESP	3,6915417	0,004750815	1,49E-04	1,7987833	-0,08544016
GPR37	3,6843379	2,19E-04	1,19E-06	0,14917104	1,1544151
CRIP2	3,6831179	0,00682715	2,52E-04	1,9767374	0,09580978
TSPAN32	3,6566956	1,09E-04	3,41E-07	2,2392664	0,36872593
CKM	3,636942	0,03820987	0,002949841	3,480128	1,6174022
PPP1R13B	3,6296182	3,60E-04	2,74E-06	1,8665076	0,006689867
RBM20	3,6170778	2,75E-05	4,88E-08	1,3341833	-0,5206413
NKX2-5	3,581766	0,002379217	5,17E-05	2,489312	0,6486408
OLFML3	3,5575848	0,001365693	2,21E-05	0,5559063	-1,2749919
MYL7	3,535973	0,013819166	7,19E-04	1,7268791	-0,095228195
FBXO32	3,5184667	2,07E-04	1,05E-06	1,6830946	-0,13185231
SLC30A3	3,5166826	0,001237455	1,89E-05	0,8049291	-1,009286
PLAT	3,4716353	0,001677683	2,93E-05	1,0712495	-0,724366
SORBS2	3,4538682	0,005852959	2,04E-04	3,016341	1,228128
SEPT5	3,4329898	0,015691271	8,55E-04	2,90956	1,1300944
C9orf61	3,409948	0,002582079	5,88E-05	2,0682795	0,29852977
NEXN	3,4092734	0,001710757	3,04E-05	1,5766221	-0,19284217
SRD5A2L2	3,3991969	0,021132408	0,001271074	2,4319484	0,6667544

MGP	3,3922176	9,19E-04	1,23E-05	2,7115831	0,94935435
FLJ14712	3,3721278	2,84E-04	1,70E-06	1,1248193	-0,62883997
PRICKLE1	3,3676193	9,42E-05	2,74E-07	0,9740052	-0,7777238
HAND2	3,3621285	1,30E-04	4,53E-07	1,6647989	-0,08457597
TBX2	3,3362136	0,004309945	1,28E-04	1,7411036	0,002892017
SLC25A4	3,3243535	0,001007757	1,41E-05	1,5023276	-0,23074627
TCAP	3,3101537	0,007200251	2,72E-04	2,7156098	0,98871166
C7	3,2976668	8,12E-04	9,98E-06	2,0937254	0,3722798
SLC7A7	3,2889786	0,003884418	1,10E-04	1,3414155	-0,37622404
MATN2	3,281803	1,17E-04	3,76E-07	1,7693001	0,054811478
SMPX	3,272016	0,045434188	0,003755015	3,0313826	1,3212028
PLN	3,2703447	0,026546106	0,001773608	2,8785164	1,1690737
KLF2	3,2662115	1,65E-04	7,01E-07	1,3378048	-0,36981344
TRIM63	3,2251358	0,005480547	1,83E-04	2,1077464	0,41838646
C11orf9	3,224255	0,008563208	3,58E-04	1,3988976	-0,2900683
LDB3	3,2203803	8,19E-04	1,02E-05	2,324396	0,6371648
GATA5	3,1914952	2,62E-05	4,39E-08	1,0712653	-0,6029671
C20orf75	3,1792085	1,78E-04	8,05E-07	0,24990559	-1,4187621
CRYAB	3,156189	6,62E-04	7,31E-06	2,3148491	0,6566655
ACTC1	3,1380687	0,02894422	0,002000163	1,8130317	0,16315477
KBTBD10	3,1290946	0,027534781	0,00186439	2,6309159	0,98517066
KCNA5	3,1244133	1,20E-04	3,89E-07	1,3408452	-0,3027401
BMP5	3,112742	0,003539423	9,61E-05	1,2279664	-0,41021952
ACTA2	3,1113927	0,00208856	4,26E-05	1,8652254	0,22766495
GUCY1A3	3,1107242	2,18E-05	3,46E-08	0,86077356	-0,08610407
ACTN2	3,1061938	0,004813284	1,52E-04	2,4146454	0,7794976
CORIN	3,0962393	0,007595151	2,98E-04	2,3456721	0,7151551
D4S234E	3,087917	0,009917023	4,45E-04	1,4979806	-0,12865336
RFTN1	3,0800111	0,00163111	2,79E-05	0,94535094	-0,67758465
BMP2	3,075213	8,60E-04	1,10E-05	0,97970706	-0,6409793
MGC16121	3,0597966	0,026655752	0,001784923	1,2005996	-0,41283622
DUSP26	3,0313241	0,003563919	9,71E-05	2,2414742	0,6415259
PTPLA	3,025042	0,019477736	0,001148013	1,3729644	-0,22399075
EMILIN2	3,0165303	9,19E-04	1,23E-05	0,8741949	-0,71869516
PDLIM3	3,004439	1,23E-04	4,12E-07	1,8297247	0,24262889
LOC100128893	3,0036888	5,02E-04	4,51E-06	0,97419006	-0,6125453
XPO4	3,002675	0,002065758	4,11E-05	1,3436704	-0,24257803
GJA3	2,991489	0,023592884	0,001490843	-1,0834988	-0,40466008
ADM	2,9866836	0,007115443	2,66E-04	0,77722025	-0,8013242
HIST2H2BE	2,9584727	5,91E-04	6,01E-06	1,7853494	0,22049682
LRRN3	2,9539225	8,86E-05	2,44E-07	1,6321349	0,06950299
POPDC2	2,9440193	0,001294584	2,02E-05	2,1099868	0,55219966
SMAD6	2,9380832	0,003452754	9,24E-05	0,6852185	-0,86965674
IRX5	2,9332623	4,63E-04	4,02E-06	1,1385933	-0,41391277
ITGA3	2,9273074	2,46E-04	1,38E-06	1,5463327	-0,003241539
GATM	2,8956273	7,47E-04	8,81E-06	1,9061679	0,3722919

WNT2	2,8809998	0,002730171	6,33E-05	1,5163163	-0,01025327
SLIT3	2,880737	7,99E-04	9,70E-06	1,35735	-0,16908805
COBLL1	2,8723943	1,04E-04	3,15E-07	0,98910016	-0,5331537

Supplemental Table 1b. Top 100 lists of downregulated transcripts, ordered by Fold Change.

Day 5: M+X+ vs M-X+, FC>1.5, P<0.05

				Normalized values:	
**Symbol**	**FC (abs)**	**Corrected p-value**	**P-value**	**[D5 M+]**	**[D5 M-]**
C9orf135	14,172698	1,30E-09	3,50E-13	0,3792173	4,20426
HRASLS3	12,230516	1,26E-09	1,44E-13	-0,36665788	3,2457554
CDH1	11,195227	2,00E-09	7,18E-13	-1,6084255	1,8763862
CAMKV	10,866297	3,11E-09	1,68E-12	-0,59813946	2,843649
SOX2	10,30234	2,49E-08	3,20E-11	-1,3072952	1,8159451
GRPR	10,199672	9,82E-09	8,82E-12	-0,18834496	1,2287785
LOC642559	9,745841	1,26E-09	1,73E-13	0,007226467	3,2920132
POU5F1P1	9,245438	8,26E-10	2,47E-14	0,11335007	3,2571964
TACSTD1	9,152361	7,32E-09	5,92E-12	-0,63080263	0,43678632
SCNN1A	8,959293	2,89E-09	1,47E-12	-0,25767088	2,905714
RPRM	8,889885	9,97E-09	9,25E-12	-0,7779393	2,3742254
KIF1A	8,79679	3,22E-08	4,63E-11	-1,2301893	1,9067878
LECT1	8,76456	1,34E-09	4,01E-13	0,050319035	3,1022785
POU5F1	8,730854	2,89E-09	1,35E-12	0,36910772	3,4952304
LOC645682	8,479693	1,27E-09	2,67E-13	0,20764144	3,2916534
GLDC	8,355546	1,30E-09	3,44E-13	-1,6761416	1,3865924
ITGB1BP3	8,166984	2,73E-08	3,74E-11	-0,21106148	1,309834
L1TD1	8,139897	1,26E-09	1,97E-13	-0,19010305	2,8349075
PDPN	8,014854	1,83E-07	4,87E-10	-0,62393713	2,378739
TFAP2A	7,892454	1,79E-08	2,14E-11	-0,6334855	0,94715977
FAM46B	7,6947074	4,10E-09	2,65E-12	-0,22864325	2,7152233
ZSCAN10	6,976572	1,29E-08	1,33E-11	0,19084628	2,9933646
EPCAM	6,938631	7,28E-09	5,44E-12	-1,3012419	1,493409
SOX21	6,853765	3,04E-07	9,93E-10	-1,1762661	1,6006306
DBNDD1	6,840434	8,89E-08	1,80E-10	-0,37628493	1,2283705
RHBDL3	6,6835656	2,73E-07	8,50E-10	-0,12929504	2,6113229
CRABP1	6,650401	2,35E-09	9,16E-13	-1,1311678	1,6022735
CDH6	6,5062366	2,89E-09	1,47E-12	-0,8197713	1,882052
SCGB3A2	6,214735	2,73E-08	3,84E-11	-0,114228405	2,5214643
ZIC2	6,005795	1,82E-05	1,71E-07	-2,4202604	0,16609478
PRDM14	5,833089	8,89E-08	1,79E-10	-0,32682577	1,3381763
CKB	5,8221173	5,69E-07	2,13E-09	-1,1681575	1,3733864
PIM2	5,755149	2,89E-09	1,42E-12	-0,048635643	2,4762175
STMN3	5,698341	8,59E-08	1,67E-10	-1,1243219	1,3862201
SMPDL3B	5,532676	1,26E-09	2,27E-13	-0,27982506	2,1881526
TUBB3	5,4109774	2,31E-07	6,77E-10	-0,58805007	1,8478392
SFRP2	5,352398	3,91E-09	2,31E-12	-1,4320765	0,98810893
CRMP1	5,334716	3,82E-07	1,28E-09	-1,1639237	1,2514876
CXCL12	5,236672	7,00E-08	1,22E-10	-0,58579844	0,9072903
LOC440132	5,178706	7,43E-08	1,35E-10	-0,10400534	2,2685864
ADM	5,113436	1,33E-06	6,35E-09	0,035577614	2,3898706

IDO1	5,0950255	9,15E-08	1,92E-10	0,03948927	2,3885787
EPHA1	5,024444	3,91E-09	2,34E-12	-0,46000162	1,8689624
TTYH1	5,0000196	2,05E-07	5,71E-10	-0,24567683	0,4162456
LOC643272	4,966123	4,35E-07	1,51E-09	0,24664657	2,5587666
PPP2R2B	4,9369545	1,35E-08	1,49E-11	-0,2648193	0,7775521
MIAT	4,872266	1,31E-05	1,12E-07	-0,5640709	1,7205219
NLGN4X	4,850735	4,94E-08	7,69E-11	-0,41810894	1,7658877
TJP3	4,8324614	1,52E-06	7,69E-09	-0,46595827	1,8068
CLDN7	4,8223386	2,73E-08	3,78E-11	-0,7868185	1,4829143
CYP2S1	4,79607	2,49E-08	3,14E-11	-0,15951411	0,6961684
PRODH	4,7019587	1,81E-06	9,43E-09	0,7477849	2,9810467
ERBB3	4,5258307	1,09E-08	1,06E-11	-1,0389463	1,1392363
PMAIP1	4,4913087	1,84E-06	9,62E-09	0,027835688	2,1949716
GABRB3	4,4657955	4,04E-08	5,92E-11	-0,2000211	1,8181845
DNMT3B	4,4336934	1,53E-08	1,74E-11	0,76085633	2,9093654
FRAT2	4,4082026	4,10E-09	2,82E-12	0,09872977	2,2389205
MAL2	4,3654065	9,65E-08	2,18E-10	-0,9914748	1,1346413
ID1	4,2613378	1,78E-07	4,63E-10	-0,42959628	0,59004116
TFAP2C	4,2300897	5,52E-07	2,05E-09	-0,72665834	1,2723951
F2RL1	4,207916	8,15E-07	3,24E-09	-0,45571232	1,3378094
INDO	4,1338515	1,66E-06	8,60E-09	-0,12049166	1,9269948
MATK	4,132926	5,31E-08	8,59E-11	-0,6254673	1,4216963
SEL1L3	4,078408	4,56E-07	1,60E-09	-1,121761	0,90624523
UGP2	4,053473	9,82E-09	8,61E-12	-0,86995727	1,1492013
IFITM1	4,0520887	7,67E-08	1,42E-10	0,27845225	2,297118
POLR3G	4,0298495	1,09E-08	1,07E-11	-0,20192559	0,62291497
LOC649970	3,9713933	1,58E-08	1,85E-11	-0,6690257	1,3206195
SCG3	3,8773568	2,47E-08	3,03E-11	-0,23751147	1,230738
FLJ22184	3,8745868	4,24E-07	1,46E-09	-1,073164	0,88087845
LOC646316	3,8086455	1,06E-05	8,51E-08	0,27052513	2,199803
CD24	3,7852619	4,52E-06	3,02E-08	0,21074772	1,1774665
NPTX2	3,7236927	7,18E-08	1,27E-10	-0,6349824	1,2617515
HRK	3,7147868	3,96E-07	1,34E-09	-0,12372112	1,7695583
LAD1	3,7054057	9,65E-08	2,18E-10	-0,5174263	1,3722053
CLDN6	3,6712868	1,40E-07	3,45E-10	-0,30945906	1,5668267
UCHL1	3,6583219	2,71E-07	8,37E-10	-0,46144614	1,4097358
ALPL	3,6211176	8,83E-07	3,71E-09	-0,54576284	1,3106722
HPGD	3,6152725	9,82E-09	8,64E-12	-0,18060382	1,6735005
DCLK1	3,6149669	1,44E-06	7,16E-09	0,06391064	1,917893
FLJ25404	3,612953	2,01E-06	1,11E-08	-0,8097372	1,0434413
TERF1	3,5710814	1,12E-05	9,29E-08	-0,117455326	0,58357716
TRIML2	3,5264497	1,58E-07	4,07E-10	-0,19940615	1,6188103
C21orf59	3,432132	7,50E-07	2,92E-09	-1,77E-04	0,6572178
RARRES2	3,422109	1,29E-08	1,36E-11	-0,8990539	0,8758318
CKMT1A	3,4140503	5,31E-08	8,47E-11	-0,6289832	1,1425012
SEMA6A	3,4036477	1,40E-06	6,81E-09	-0,10901912	1,6580626

LOC284620	3,4023583	4,21E-07	1,44E-09	-0,21105178	1,5554832
ENO2	3,400855	2,13E-06	1,21E-08	-0,82657194	0,9393255
ZNF296	3,3651702	1,04E-07	2,41E-10	-0,05553007	1,6951494
TMEM125	3,3567498	4,37E-08	6,61E-11	-0,38707733	1,3599876
PODXL	3,3485131	4,37E-08	6,66E-11	-0,28997183	1,084808
CABP7	3,3458052	1,11E-07	2,61E-10	-0,050436813	1,6919166
FOXO1	3,3426983	1,88E-07	5,07E-10	-0,087767124	1,653246
RND2	3,3164933	2,83E-06	1,70E-08	-0,3204395	1,4092191
BCL11A	3,316361	1,23E-07	2,98E-10	-0,72589254	1,0129493
LOC646817	3,2931757	9,16E-06	7,05E-08	0,08722496	1,8067044
TMEM64	3,2822366	1,55E-07	3,95E-10	-0,043886345	1,6707929
PPAP2A	3,2676947	1,25E-06	5,89E-09	-0,08295647	1,2027096
HTR3A	3,2649605	8,59E-08	1,65E-10	-0,14979441	1,5572711

Day 7: M+X+ vs M-X+, FC>1.5, P<0.05

				**Normalized values:**	
**Symbol**	**FC (abs)**	**Corrected p-value**	**P-value**	**[D7 M+]**	**[D7 M-]**
ACPP	2,7686253	0,00463394	2,93E-05	0,2926472	1,7618171
ADM	2,7891867	0,003548604	2,03E-05	-1,1627827	0,3170619
AIF1L	3,345002	0,001516203	5,51E-06	-1,2083424	0,5213906
ALDH4A1	1,9533156	0,02713714	4,59E-04	0,071024895	0,7372999
ALPL	4,826471	2,64E-05	4,25E-09	-1,9109892	0,35997948
ARHGEF19	2,9934182	1,52E-04	8,64E-08	-0,92695934	0,65483457
ASNS	2,8057985	0,004841323	3,22E-05	-0,85569125	0,6327202
ASS1	2,5547225	0,005070563	3,51E-05	-1,4285756	0,31936947
ASTN2	2,03794	0,009129052	8,36E-05	-0,15893523	0,8681763
AUTS2	2,5663798	8,85E-04	2,60E-06	-0,36578956	0,9939451
BNIPL	5,224844	8,66E-04	2,46E-06	-0,257861	2,127527
C1orf115	2,0625703	0,004788261	3,11E-05	-0,30635262	0,7380908
C9orf58	2,835225	0,011927551	1,30E-04	-0,66817313	0,83529013
CA12	2,1337938	0,040067665	8,85E-04	0,15755367	1,2509745
CAMK2N1	2,11352	0,009280975	8,58E-05	-0,25667715	0,8229707
CAMKV	4,606133	2,02E-04	1,36E-07	-0,81662226	1,3869339
CBS	2,3540192	0,002648344	1,39E-05	-1,1836596	0,051466465
CCND1	2,2423809	0,040866632	9,15E-04	-1,5114765	-0,34644523
CD24	2,503957	4,79E-04	8,18E-07	-0,042955875	0,70667773
CDH1	7,0339627	5,72E-04	1,14E-06	-0,8550477	1,9592899
CDH6	2,181875	3,40E-04	3,99E-07	-0,53789586	0,58767253
CDT1	2,4467359	0,002675488	1,43E-05	-1,12176	0,16909838
CEBPB	2,3932574	0,022296214	3,48E-04	-0,7495168	0,50945884
CGN	3,8853197	2,59E-04	2,79E-07	-0,507312	1,4507214
CLDN10	8,954492	0,002046865	8,87E-06	0,22938776	3,1392746
CLDN6	2,4757056	0,002900348	1,59E-05	-0,101872765	1,205967
CLDN7	3,5332584	4,13E-04	5,89E-07	-0,7351208	1,0858785
CNTNAP2	2,1074705	0,008048192	6,91E-05	-1,8575228	-0,7820104
COBL	2,3682604	0,02791715	4,78E-04	-0,111570835	1,1322569

CRABP1	2,4630663	2,59E-04	2,66E-07	-1,753119	-0,45266357
CRMP1	3,7356668	0,002873934	1,57E-05	-1,664701	0,23666477
CYP1B1	3,5447168	8,90E-04	2,66E-06	-0,73781586	1,0878545
CYP26A1	5,5871043	8,53E-04	2,36E-06	-0,9291398	1,552961
CYP2S1	2,374267	0,008345273	7,32E-05	-0,5200235	0,7274588
DBI	2,6848304	4,59E-04	7,40E-07	-0,6390381	0,55039626
DBNDD1	4,313914	2,92E-05	6,47E-09	-1,6981155	0,41088185
DHCR24	2,1405823	0,034103826	6,83E-04	-1,1167	-0,018696627
DIAPH2	2,0826068	0,039393436	8,61E-04	-0,39520612	0,66318434
DKK3	2,1720297	0,03254569	6,37E-04	-0,746648	0,37239584
DLL1	2,8792908	0,011748497	1,27E-04	-0,50417185	1,0215417
DLX3	2,0325708	0,009057742	8,14E-05	-0,08511734	0,9381884
DLX5	6,768752	8,32E-04	2,17E-06	0,13308239	2,8919723
DNMT3B	3,9140496	0,002119495	9,33E-06	-0,61933297	1,1387644
DPEP3	2,0775878	0,005771553	4,42E-05	-0,20667362	0,8482359
DPPA4	2,1768997	0,002585767	1,31E-05	-0,5771839	0,545091
EDN1	1,9867797	6,98E-04	1,65E-06	-0,17255433	0,8178776
EDNRB	3,1704311	0,018824795	2,65E-04	-0,11584028	1,5488387
EFHD1	2,685918	0,023703383	3,77E-04	-0,26070896	1,1647063
EFNA1	2,1916358	0,009057742	8,16E-05	0,04625845	0,6848378
EPAS1	6,2133536	0,00700534	5,81E-05	0,22862689	2,8639991
EPCAM	6,5609984	3,63E-05	9,77E-09	-0,9078981	1,8060173
EPHA1	3,2740517	0,001315298	4,49E-06	-0,54991055	1,1611665
ERBB3	2,508194	5,32E-04	9,71E-07	-0,81998205	0,50666684
F11R	2,2999327	0,00930231	8,66E-05	-0,26367807	0,9379136
F2RL1	2,2624366	0,003844972	2,27E-05	-0,25064006	0,85071516
FAM123A	3,6637661	0,008252493	7,21E-05	0,12680657	2,000134
FAM46B	3,7103908	6,63E-04	1,55E-06	-0,2632246	1,6283466
FHDC1	2,0294201	0,03936264	8,58E-04	-0,36861482	0,65245277
FLJ22184	2,4754157	8,68E-04	2,49E-06	-0,5847287	0,7229422
FLJ25404	2,130426	4,65E-04	7,80E-07	-1,0233202	0,06782166
FNBP1L	2,1293182	0,01475009	1,82E-04	-0,74148065	0,34891096
FRAT2	4,0025043	8,37E-04	2,20E-06	-0,5889948	1,4119081
GABRB3	2,0761373	0,001516203	5,54E-06	-0,08288447	0,6761176
GABRP	3,5437038	0,029978584	5,49E-04	0,6086958	2,4339538
GJA1	4,148553	0,001221311	4,13E-06	-1,6008244	0,45178398
GLDC	3,53191	0,00326204	1,83E-05	-1,6748238	0,1456248
GPR56	2,3223734	0,015985066	2,08E-04	-0,38115963	0,8344404
GRPR	2,4567263	0,02102351	3,17E-04	-0,006062031	1,2906752
HACL1	2,3783653	7,63E-04	1,85E-06	-1,0983634	0,15160687
HPGD	3,6436896	0,001694897	6,75E-06	-0,04384168	1,8215584
HRASLS3	3,0866513	0,003133927	1,74E-05	-0,25749016	1,3685523
ID1	2,376764	0,005005242	3,37E-05	0,1278553	1,3768541
IFITM1	2,001297	0,004219235	2,58E-05	-0,9519318	0,0490036
IGSF1	2,2108989	0,012412748	1,40E-04	-0,34639263	0,79824036
IRX2	2,4666018	0,01959785	2,82E-04	-0,46715036	0,83537453

ITGB1BP3	2,3834648	0,03048775	5,67E-04	-0,5403061	0,71275425
ITPR3	2,1554308	0,001341349	4,66E-06	-0,81079197	0,29718432
KCNK12	16,270681	2,58E-06	7,72E-11	-0,9335639	3,090639
KHDRBS3	2,8009512	5,72E-04	1,15E-06	-0,34699678	0,47477117
KIF1A	3,5147471	0,001098713	3,61E-06	-1,7354409	0,07798004
KIT	2,6845145	0,010971687	1,14E-04	-0,19821899	0,76878375
KRTAP21-1	2,453022	4,59E-04	7,43E-07	0,25532547	1,5498856
KRTAP21-2	3,039406	4,03E-04	5,19E-07	0,42285457	2,026644
KRTAP6-3	3,0989351	2,59E-04	2,52E-07	0,33275428	1,9645268
L1TD1	7,565453	2,02E-04	1,39E-07	-0,83095676	2,0884697
LAD1	2,5693686	5,49E-04	1,05E-06	-0,53434867	0,82706517
LECT1	2,3321831	0,002455001	1,21E-05	-0,19703484	0,81060773
LFNG	2,1695235	2,25E-04	1,62E-07	-1,2349938	-0,11761554
LIN28	2,5900643	7,65E-04	1,92E-06	-1,1659261	0,20706177
LINGO1	2,3658576	5,72E-04	1,14E-06	-0,5299471	0,7124162
LITAF	2,3785443	0,002330476	1,11E-05	-1,0756284	0,17445056
LOC284620	2,4919174	0,001028323	3,23E-06	-0,12888654	1,1883696
LOC387763	2,1277454	6,13E-04	1,28E-06	-0,13816054	0,951165
LOC440132	2,412278	4,79E-04	8,31E-07	-0,2130359	1,0573602
LOC441506	1,9878395	0,001694897	6,68E-06	-0,79103214	0,20016909
LOC642559	10,132142	4,11E-04	5,41E-07	-1,7976707	1,5431966
LOC645682	4,161805	2,28E-04	1,71E-07	-0,69322664	1,3639828
LOC646817	2,722062	5,52E-05	2,28E-08	-0,6661668	0,7785333
LOC647262	2,0868776	9,95E-04	3,07E-06	0,17331123	1,2346573
LOC647264	2,4124365	2,40E-04	2,07E-07	0,116141796	1,3866328

Day 10: M+X+ vs M-X+, FC>1.5, P<0.05

				**Normalized values:**	
**Symbol**	**FC (abs)**	**Corrected p-value**	**P-value**	**[D10 M+]**	**[D10 M-]**
ZIC2	11,356423	4,79E-11	1,43E-15	-2,1904953	1,3149414
ALPL	8,352684	2,82E-10	3,38E-14	-2,3192914	0,74294853
SOX21	7,1928244	3,97E-09	2,26E-12	-0,9096481	1,9369102
SOX2	6,9907885	1,19E-07	1,32E-10	-0,9874094	1,7527102
RAX	6,4284477	2,82E-10	2,79E-14	0,20175886	2,8862293
LIN28	6,179589	4,61E-10	1,52E-13	-2,6252174	0,002293428
BTBD17	5,6779304	3,56E-10	8,56E-14	-0,17541313	2,329952
FABP7	5,662112	3,52E-10	5,26E-14	-0,3332804	2,16806
SFRP2	5,656748	3,56E-10	9,06E-14	-0,7725914	1,7273813
CNTNAP2	5,5924544	9,86E-10	3,84E-13	-2,2682083	0,21527338
VTCN1	5,4066296	1,01E-04	1,16E-06	0,41458115	2,8493106
KCNK12	5,386756	8,62E-10	3,10E-13	-1,4000672	1,0293494
TFAP2A	5,339943	3,38E-07	5,47E-10	-0,08843056	1,4349962
CRABP1	4,9853168	3,56E-10	9,59E-14	-1,2938014	1,0238837
PAMR1	4,885397	3,83E-09	2,07E-12	-0,20570739	2,0827684
HESX1	4,6827188	1,09E-05	5,91E-08	-0,01464653	2,2127
GABRP	4,59971	0,005285287	2,05E-04	1,2949816	3,4965246

CDH6	4,5083604	3,54E-10	6,35E-14	-0,17672713	1,9958758
SP8	4,256085	3,62E-10	1,08E-13	-0,064917244	0,96517164
TGFBI	4,2264657	1,73E-05	1,13E-07	-0,39719138	1,6822604
CRMP1	4,1242404	1,48E-09	6,66E-13	-1,4955045	0,54862386
PPP2R2B	4,106863	4,46E-08	3,47E-11	-0,11498944	0,5694507
FLJ22184	4,002066	3,02E-08	1,81E-11	-0,20582835	1,7949167
LFNG	3,995137	1,48E-09	6,54E-13	-1,1090118	0,8892333
GLDC	3,9873145	9,27E-08	9,71E-11	-0,22237189	-1,0614172
CAMKV	3,986148	4,97E-08	4,17E-11	-1,0309306	0,9640648
PRSS8	3,9741106	7,97E-08	7,71E-11	-1,0085088	0,9821232
SOX3	3,9703567	4,14E-08	3,10E-11	-0,2866877	1,7025809
AIF1L	3,8764882	5,37E-08	4,66E-11	-1,337686	0,23137617
L1TD1	3,8371923	3,52E-07	5,87E-10	-1,2687874	0,6712637
TTYH1	3,7783687	2,35E-09	1,20E-12	-0,35221162	1,5655519
CDH1	3,7284539	5,04E-04	9,45E-06	-1,0007234	0,89785403
FLJ25404	3,6757991	9,24E-08	9,40E-11	-0,99893886	0,8791191
SIX3	3,6731842	6,69E-07	1,27E-09	0,14118798	2,0182192
PAX6	3,6590078	2,35E-09	1,17E-12	0,14994843	1,31449
DNMT3B	3,6303434	6,40E-07	1,19E-09	-1,0721477	0,30256638
KIAA0101	3,541487	4,80E-08	3,88E-11	-0,46870613	0,12671645
GJA1	3,4696815	1,84E-06	5,24E-09	1,8953596	0,441535
ERP27	3,43251	3,07E-06	9,63E-09	0,42479977	2,2040637
CGN	3,35595	7,93E-07	1,61E-09	-0,6516757	1,0950456
DBNDD1	3,3539724	4,14E-08	2,99E-11	-1,3384589	0,4074119
LOC728715	3,348329	2,26E-07	3,11E-10	-0,8996382	0,8438032
DLX5	3,3328238	3,30E-07	5,23E-10	0,17037487	1,9071199
OTX2	3,3266892	1,24E-07	1,41E-10	-0,12324842	1,2399069
HES5	3,3065825	5,69E-06	2,32E-08	-0,47523975	1,2501011
MIAT	3,276623	1,18E-07	1,27E-10	-0,45281473	1,259395
RGMA	3,2634118	9,29E-07	2,09E-09	-0,83266973	0,8737113
STOM	3,251944	4,81E-06	1,89E-08	-0,17260201	0,996264
AFP	3,216727	1,80E-05	1,18E-07	0,14387195	1,8294654
TMSB15A	3,1780186	1,44E-07	1,72E-10	-1,0670458	0,60108167
ZFP42	3,1559532	1,21E-06	3,00E-09	-1,4463233	0,21175258
LHX2	3,1274269	4,35E-07	7,68E-10	0,56345844	2,2084346
FJX1	3,115349	1,84E-06	5,27E-09	-0,06903219	1,5703616
CCNB2	3,0930676	1,59E-06	4,28E-09	-1,8379706	-0,20893224
UBE2C	3,0567656	7,02E-06	3,27E-08	-1,6226779	-0,049807865
PSAT1	3,0484145	5,42E-08	4,87E-11	-1,4160709	0,19198816
BNIPL	3,0254493	3,52E-07	5,90E-10	-0,26417017	1,3329792
CDC20	3,0142345	4,20E-06	1,48E-08	-1,6940175	-0,10222594
CYP26A1	3,0063374	5,82E-04	1,14E-05	-0,9882596	0,59974736
ZSCAN10	2,9751365	4,24E-06	1,55E-08	-0,990355	0,5826008
APOA2	2,9611192	7,59E-05	7,97E-07	-0,32042995	1,2457126
FAM64A	2,9532187	5,73E-08	5,32E-11	-1,4688511	0,09343704
KIF1A	2,943759	2,12E-07	2,85E-10	-1,0709914	0,4866681

TACSTD2	2,940513	5,85E-06	2,45E-08	0,3893261	1,9453939
TFAP2C	2,9399054	6,69E-07	1,28E-09	-0,6136217	0,6266632
SALL4	2,892518	3,40E-04	5,61E-06	-1,3414956	0,1908304
CKS2	2,8753078	7,93E-07	1,61E-09	-1,5770946	-0,14751911
ARHGEF19	2,8523476	7,97E-08	7,88E-11	-0,7637326	0,7484172
EPAS1	2,8429148	0,022822592	0,001351306	0,3807478	1,8881186
RAB25	2,8411336	8,17E-07	1,69E-09	-0,64916563	0,85730106
TUBB2B	2,838742	4,90E-05	4,14E-07	-1,2664323	0,23881944
CBS	2,835333	1,59E-06	4,25E-09	-1,6773248	-0,1738065
GINS2	2,8110423	1,83E-06	5,14E-09	-0,69793415	-0,07470226
SOX15	2,7925508	1,08E-06	2,52E-09	-0,006700357	1,4748831
LOC731932	2,7771945	6,25E-06	2,75E-08	-0,050079346	1,4235488
MGST1	2,7760875	4,71E-06	1,82E-08	-1,4789096	-0,07707945
NCAPG	2,7705257	8,54E-06	4,17E-08	-1,4575261	0,012633641
NUSAP1	2,7590625	4,25E-06	1,59E-08	-1,1596155	-0,10244894
OIP5	2,742401	3,76E-06	1,28E-08	-0,95553714	-0,10998821
UHRF1	2,7318711	4,80E-05	4,04E-07	-1,7387415	-0,28885207
LOC642559	2,7275486	6,07E-04	1,22E-05	-2,362481	-0,91487616
TOP2A	2,7030442	9,73E-05	1,10E-06	-1,7057587	-0,27117363
ANKS1A	2,651612	3,73E-04	6,37E-06	-0,17984946	1,2270203
DLGAP5	2,6507142	2,17E-05	1,47E-07	-1,3518677	-0,002206643
TUBA1C	2,650279	1,73E-05	1,12E-07	-0,91921884	-0,1061875
SEL1L3	2,6273944	3,21E-07	5,00E-10	-0,5211393	0,87249345
PRIM1	2,6164975	3,83E-06	1,32E-08	-1,4723102	-0,18334071
SLC35F2	2,6097214	1,67E-04	2,21E-06	-0,8132267	0,19904725
LMO1	2,6070075	1,78E-04	2,40E-06	0,06277832	1,4451731
CENPN	2,598287	5,93E-06	2,52E-08	-1,5710245	-0,19346364
C9orf58	2,5976982	6,08E-06	2,60E-08	-0,9864432	0,3907906
DNMT3B	2,5931652	4,47E-05	3,65E-07	-1,652762	0,20734389
CDCA5	2,5866795	2,26E-05	1,57E-07	-1,6079965	-0,23689525
CDT1	2,5778306	4,24E-06	1,53E-08	-1,4316119	-0,06545448
CENPV	2,575101	1,36E-06	3,48E-09	-0,7888196	0,34394312
CAPN6	2,5350916	3,21E-06	1,02E-08	0,05508105	1,3971189
VWF	2,5304518	1,32E-05	7,62E-08	0,17454736	1,5139422
KANK4	2,4842618	0,002130468	6,26E-05	0,44607162	1,7588888
TLE4	2,4757755	2,90E-04	4,56E-06	-0,071427025	0,69211245
LOC729779	2,4630144	3,81E-05	2,94E-07	-1,2888474	0,011577606

Day 14: M+X+ vs M-X+, FC>1.5, P<0.05

				**Normalized values:**	
**Symbol**	**FC (abs)**	**Corrected p-value**	**P-value**	**[D14 M+]**	**[D14 M-]**
HES5	20,32905	9,91E-08	2,97E-12	0,44802204	4,793493
ZIC2	9,244635	1,83E-06	6,03E-10	-1,8858725	1,3227439
LIN28	8,470935	1,15E-05	1,13E-08	-3,7302542	-0,64773303
SOX21	8,354947	6,14E-05	1,51E-07	-0,79964525	2,2629855
FABP7	8,197753	1,83E-06	5,49E-10	-0,4370602	2,5981684

SOX2	7,8771815	3,50E-04	2,60E-06	-0,5745039	2,4031756
MIAT	7,351799	1,92E-05	2,88E-08	0,041386604	2,919484
LOC650757	7,3392096	6,88E-07	8,45E-11	0,2970141	3,1726387
RAX	7,296329	1,04E-05	9,62E-09	1,4067574	4,273928
SOX3	6,6596613	2,41E-07	1,44E-11	0,1740923	2,9095411
LFNG	6,4692984	9,31E-07	1,95E-10	-0,77050114	1,9231082
CNTNAP2	6,091239	6,88E-07	6,67E-11	-2,5871384	0,019597372
SP8	5,9891577	4,80E-06	2,32E-09	-0,24506664	2,3372865
ALPL	5,904092	1,20E-05	1,40E-08	-2,6507986	-0,089083515
TTYH1	5,869242	6,88E-07	1,03E-10	-0,053366978	2,4998074
BTBD17	5,7780585	5,52E-06	2,98E-09	0,21714084	2,7477257
CRABP1	5,7497177	7,43E-06	4,89E-09	0,77556896	3,29906
PAMR1	5,61678	1,23E-04	4,15E-07	-0,2918973	2,197846
C18orf51	5,5560646	8,18E-06	5,88E-09	0,51417685	2,9882402
PAX6	5,4933634	1,55E-05	2,14E-08	0,8536639	3,3113537
SIX6	5,4576697	9,42E-06	7,89E-09	1,3294138	3,7776988
C18orf51	5,316013	1,83E-06	4,75E-10	0,3295566	2,7399013
ALDH1A3	5,219608	6,35E-06	3,61E-09	0,31717524	2,7011166
GLDC	4,9462657	2,64E-06	1,03E-09	0,22300608	-0,9531372
OTX2	4,715546	2,42E-04	1,34E-06	-1,0542421	1,1831826
LHX2	4,714058	1,57E-05	2,21E-08	0,8913663	3,1283357
TMSB15A	4,656853	1,04E-05	9,44E-09	-0,9297692	1,2895861
NELL2	4,6184297	6,54E-06	3,92E-09	-0,82807463	1,3793278
CDH6	4,291971	2,43E-06	8,73E-10	0,001482487	2,103123
PTPRZ1	4,2460823	3,12E-05	6,51E-08	-0,65511674	1,4310156
SFRP2	4,197889	8,12E-04	9,95E-06	1,4914635	3,5611274
TFAP2A	4,033954	2,96E-05	5,62E-08	-0,77647877	1,2357157
PPP2R2B	3,8929794	0,002495424	5,56E-05	0,022147974	1,9830227
EMX2	3,8676414	2,37E-04	1,30E-06	0,403756	2,35521
FLJ22184	3,7990577	6,94E-05	1,81E-07	0,06393385	1,9895755
CHX10	3,7473567	3,96E-04	3,21E-06	0,86958027	2,7754536
SIX3	3,739915	2,86E-04	1,82E-06	0,435047	2,3380525
DLL1	3,7098415	8,18E-06	6,12E-09	-0,4811821	1,4101754
GAS1	3,6649065	1,20E-04	3,91E-07	-0,16575861	1,7080178
SALL4	3,60439	1,12E-04	3,51E-07	-1,9317287	-0,08197371
NUSAP1	3,5681043	1,20E-04	3,99E-07	-1,7247843	0,1103735
KIF5C	3,5066633	8,94E-06	7,02E-09	-1,0211157	0,78898317
TLE4	3,5057356	4,80E-06	2,44E-09	-0,82240885	0,9873083
GJA1	3,4630764	1,54E-05	2,08E-08	2,5277295	0,94686586
SILV	3,3339899	1,91E-05	2,79E-08	-0,9192764	0,8179733
AIF1L	3,3304207	2,11E-05	3,28E-08	-1,8634981	-0,12779362
LMO3	3,29013	8,94E-06	7,22E-09	0,39979187	2,1179364
CENPV	3,278227	1,40E-05	1,80E-08	-0,82756776	0,885348
ATP6V1B1	3,27103	3,39E-05	7,40E-08	-0,4346191	1,2751259
TUBB2B	3,2660096	9,11E-04	1,21E-05	-1,1114097	0,5961194
EFHD1	3,2401218	1,47E-05	1,93E-08	-0,74310464	0,9529433

CAMKV	3,2083385	6,56E-06	4,12E-09	-0,92221117	0,75961524
LRRC45	3,1720266	1,20E-05	1,37E-08	0,14209986	1,8075048
CCNB2	3,1596804	3,49E-04	2,57E-06	-1,8099517	-0,15017302
CRMP1	3,1585088	3,62E-05	8,12E-08	-1,3083471	0,3508965
PSAT1	3,1342695	1,30E-05	1,56E-08	-1,7236704	-0,075541176
NOS2A	3,1291807	3,09E-05	6,19E-08	-0,02383391	1,6219511
CKS2	3,094429	3,01E-04	1,99E-06	-1,5171171	0,11255614
CCL2	3,0731633	0,005227452	1,72E-04	1,0743676	2,694092
MPG	3,0635056	1,92E-05	2,93E-08	0,12164625	1,7368298
UBE2C	3,0570579	2,14E-04	1,12E-06	-1,5012969	0,110847
ZIC3	3,0528562	3,04E-05	5,92E-08	-1,3260025	0,28415713
CCKBR	3,0364652	0,011397141	5,43E-04	0,14012893	1,7425218
LIN28B	3,026221	5,05E-05	1,19E-07	-1,7169532	-0,11943579
CDC20	3,025173	2,18E-04	1,18E-06	-1,7075458	-0,11052815
ZBTB16	3,0200615	3,62E-05	8,09E-08	0,25958714	1,8541651
LRRN1	3,0025437	8,99E-04	1,18E-05	-0,90500957	0,6811757
TCEAL2	2,9847372	1,54E-04	6,24E-07	-0,43879652	1,1388074
UHRF1	2,9439883	9,27E-04	1,27E-05	-1,8227922	-0,26502022
FAM64A	2,9383962	1,20E-05	1,39E-08	-1,4758444	0,07918453
WDR54	2,9361308	0,001710757	3,03E-05	-0,81962234	0,73429394
C20orf103	2,9351726	3,10E-05	6,32E-08	0,07118845	1,6246338
SALL2	2,9348423	1,23E-04	4,19E-07	-1,0051652	0,5481178
RGMA	2,9330187	1,45E-04	5,46E-07	-1,0729852	0,4794011
CLDN1	2,914592	0,032088853	0,002311785	0,18117015	1,724464
CENPF	2,9139433	1,45E-04	5,56E-07	-1,1446873	0,39828554
SYTL2	2,9130218	2,73E-05	4,74E-08	0,23693196	1,7794484
MAD2L1	2,8921025	7,19E-04	8,35E-06	-1,4750443	0,057074387
DRD4	2,8841324	1,02E-05	8,81E-09	-0,08087317	1,4472643
LOC728715	2,8788831	8,62E-04	1,11E-05	-1,000337	0,52517223
FLJ25404	2,8773823	6,19E-05	1,54E-07	-0,9979847	0,5267722
EFEMP1	2,8678486	6,19E-04	6,47E-06	0,10570303	1,6256719
TOP2A	2,8474207	1,36E-04	4,95E-07	-1,5492262	-0,03957049
TPBG	2,845888	5,35E-04	5,15E-06	-0,117841244	1,3910376
MGC61598	2,84096	1,36E-05	1,71E-08	-0,6412222	0,86515635
MGC39900	2,8284621	1,78E-04	7,99E-07	-0,72060555	0,77941227
GINS2	2,8253868	1,60E-04	6,70E-07	-0,7683797	-0,001199881
TAGLN3	2,8182871	6,89E-05	1,75E-07	0,058712166	1,5535307
DNMT3B	2,8079605	2,29E-04	1,25E-06	-1,8951153	-0,4055926
TUBB3	2,794116	0,001026355	1,45E-05	-0,34778848	1,1346035
LOC401056	2,7919347	1,18E-05	1,21E-08	0,0704759	1,5517411
GPM6B	2,7817001	8,18E-06	6,02E-09	-0,34834543	0,26018968
FLRT3	2,7512093	1,54E-04	6,32E-07	-0,23737653	1,2226893
HMGB2	2,7381027	0,002897214	7,07E-05	-1,286182	0,16699441
CD200R1	2,7369137	1,18E-05	1,24E-08	0,20930164	1,6618515
OIP5	2,7175949	3,03E-04	2,01E-06	-1,6707745	-0,2284441
PLP1	2,7172005	3,56E-04	2,70E-06	-0,67165565	0,7704654

SCD5	2,7017329	3,17E-05	6,75E-08	0,37914768	1,8130326
PCSK5	2,699655	2,46E-04	1,39E-06	-0,07913828	1,3536369
RNF175	2,6955605	9,11E-05	2,57E-07	-0,001121521	1,4294637

Supplemental Table 2.

KEGG Pathway Analysis of enriched transcripts in MESP1+derivatives vs MESP1-derivatives (P<0.05, FC>1.5)

Day 5

Term	Count	%	PValue
hsa04510:Focal adhesion	15	3,47	8,65E-04
hsa05200:Pathways in cancer	18	4,17	0,006091023
hsa04916:Melanogenesis	8	1,85	0,016183313
hsa04310:Wnt signaling pathway	10	2,31	0,019168997
hsa05222:Small cell lung cancer	7	1,62	0,024227548
hsa04512:ECM-receptor interaction	7	1,62	0,024227548
hsa04020:Calcium signaling pathway	10	2,31	0,045200557

Day 7

Term	Count	%	PValue
hsa04512:ECM-receptor interaction	7	4,49	1,34E-04
hsa04510:Focal adhesion	8	5,13	0,002841914
hsa04310:Wnt signaling pathway	6	3,85	0,014397609
hsa05210:Colorectal cancer	4	2,56	0,046498965
hsa04350:TGF-beta signaling pathway	4	2,5641	0,050708652

Day 10

Term	Count	%	PValue
hsa05410:Hypertrophic cardiomyopathy (HCM)	20	0,28	1,18E-09
hsa05414:Dilated cardiomyopathy	20	0,28	4,90E-09
hsa04260:Cardiac muscle contraction	16	0,22	5,84E-07
hsa04510:Focal adhesion	20	0,28	7,05E-04
hsa05412:Arrhythmogenic right ventricular cardiomyopathy (ARVC)	10	0,14	0,004285607
hsa00500:Starch and sucrose metabolism	7	0,10	0,00763778
hsa04512:ECM-receptor interaction	9	0,13	0,02412758
hsa00010:Glycolysis / Gluconeogenesis	7	0,10	0,039131615
hsa00020:Citrate cycle (TCA cycle)	5	0,07	0,039392285

Day 14

Term	Count	%	PValue
hsa05410:Hypertrophic cardiomyopathy (HCM)	24	0,28	1,02E-11
hsa05414:Dilated cardiomyopathy	24	0,28	6,05E-11
hsa04260:Cardiac muscle contraction	19	0,22	3,13E-08
hsa04510:Focal adhesion	29	0,34	5,67E-07
hsa04512:ECM-receptor interaction	16	0,19	1,39E-05
hsa05412:Arrhythmogenic right ventricular cardiomyopathy (ARVC)	14	0,16	8,36E-05
hsa05200:Pathways in cancer	31	0,36	8,31E-04
hsa05212:Pancreatic cancer	10	0,12	0,009272038
hsa05012:Parkinson's disease	13	0,15	0,02602573
hsa04350:TGF-beta signaling pathway	10	0,12	0,029218018
hsa04142:Lysosome	12	0,14	0,031750928
